# Suppressor mutations in ribosomal proteins and FliY restore *Bacillus subtilis* swarming motility in the absence of EF-P

**DOI:** 10.1371/journal.pgen.1008179

**Published:** 2019-06-25

**Authors:** Katherine R. Hummels, Daniel B. Kearns

**Affiliations:** Department of Biology, Indiana University, Bloomington, Indiana, United States of America; University of Toronto, CANADA

## Abstract

Translation elongation factor P (EF-P) alleviates ribosome pausing at a subset of motifs encoding consecutive proline residues, and is required for growth in many organisms. Here we show that *Bacillus subtilis* EF-P also alleviates ribosome pausing at sequences encoding tandem prolines and ribosomes paused within several essential genes without a corresponding growth defect in an *efp* mutant. The *B*. *subtilis efp* mutant is instead impaired for flagellar biosynthesis which results in the abrogation of a form of motility called swarming. We isolate swarming suppressors of *efp* and identify mutations in 8 genes that suppressed the *efp* mutant swarming defect, many of which encode conserved ribosomal proteins or ribosome-associated factors. One mutation abolished a translational pause site within the flagellar C-ring component FliY to increase flagellar number and restore swarming motility in the absence of EF-P. Our data support a model wherein EF-P-alleviation of ribosome pausing may be particularly important for macromolecular assemblies like the flagellum that require precise protein stoichiometries.

## Author summary

Translation elongation factor P (EF-P) is a highly conserved protein that alleviates ribosome pausing at consecutive proline residues. Unlike most organisms, EF-P in the bacterium *Bacillus subtilis* is not required for growth but is instead required for a flagellar-mediated form of motility called swarming. By mapping spontaneous suppressors, we identify 7 broadly distributed ribosome-associated factors that, when mutated, allow swarming in the absence of EF-P, the location of which may provide mechanistic insight. Moreover, we show that EF-P enhances flagellar biosynthesis by alleviating ribosome pausing within a single flagellar structural component FliY and we implicate the RNA polymerase pausing factor NusG in long operon expression. Finally, we extend ribosome profiling analysis in the absence of EF-P to gram-positive bacteria.

## Introduction

Translation elongation factor P (EF-P) has been shown to alleviate ribosome pausing at consecutive proline residues (XPPX motifs) in Bacteria and Eukaryotes [1–3]. While EF-P is widely distributed and often required for rapid growth, the reason it is highly conserved is unknown [4–6]. In *Escherichia coli*, the manner in which EF-P promotes growth is thought to be pleiotropic by enhancing translation of multiple proteins that contain XPPX motifs [7]. Systems-level approaches, however, show that not all XPPX motifs induce ribosome pausing in the absence of EF-P, and that even fewer of those pauses result in decreased protein expression [8–10]. Thus, EF-P pleiotropy may be limited. Consistent with limited pleiotropy, the phenotypes of an *efp* mutant in *E*. *coli* are conditional, and are suppressed when translation rates are reduced [11]. Finally, apparent pleiotropy is organism-specific as growth defects in *Bacillus subtilis efp* mutants are negligible, even under conditions of high translation [12]. Instead, EF-P in *B*. *subtilis* is specifically required for swarming motility [12,13].

Swarming motility is a flagellar-mediated form of movement on surfaces and often requires an increase in flagellar number relative to swimming in liquid [14–18]. Increasing flagellar number is complicated as flagella are trans-envelope nanomachines that require hierarchical assembly of dozens of subunits in precise stoichiometry [19,20]. At the core of each flagellum is a type III secretion apparatus and early-class secretion is activated after the flagellar base plate and C-ring rotor are fully assembled [21–24]. Early class secreted products span the cell envelope to form an axle-like rod and universal joint-like hook [25–27]. Once the hook is polymerized to a certain length, the secretion specificity changes, the late-class sigma factor σ^D^ is activated, and late-class flagellar proteins are exported to assemble the filament [28–30]. Mutants that decrease the efficiency of flagellar expression or assembly abolish swarming motility and can do so at any step in the hierarchy [24,31]. The mechanism by which EF-P specifically activates swarming motility in *B*. *subtilis* is unknown.

Here we show that *B*. *subtilis* EF-P functions in a manner similar to that reported in other organisms and alleviates ribosome pausing at a subset of XPPX motifs. Flagellar assembly requires translation of a large number of proteins, and *efp* mutants were found to have a reduced number of flagella. Cells lacking EF-P were defective in hook completion due to translational pausing at one particular XPPX motif within the basal body component FliY. FliY in turn was necessary to activate early-class secretion. EF-P structurally resembles a tRNA and while it is thought to promote translation entropically, the mechanism is poorly understood [32]. Genetic analysis reported here further indicates that mutations in a wide variety of conserved genes related to the ribosome suppress the absence of EF-P, which may aid in the understanding of the EF-P mechanism.

## Results

### EF-P increases flagellar number

**Fig 1:**
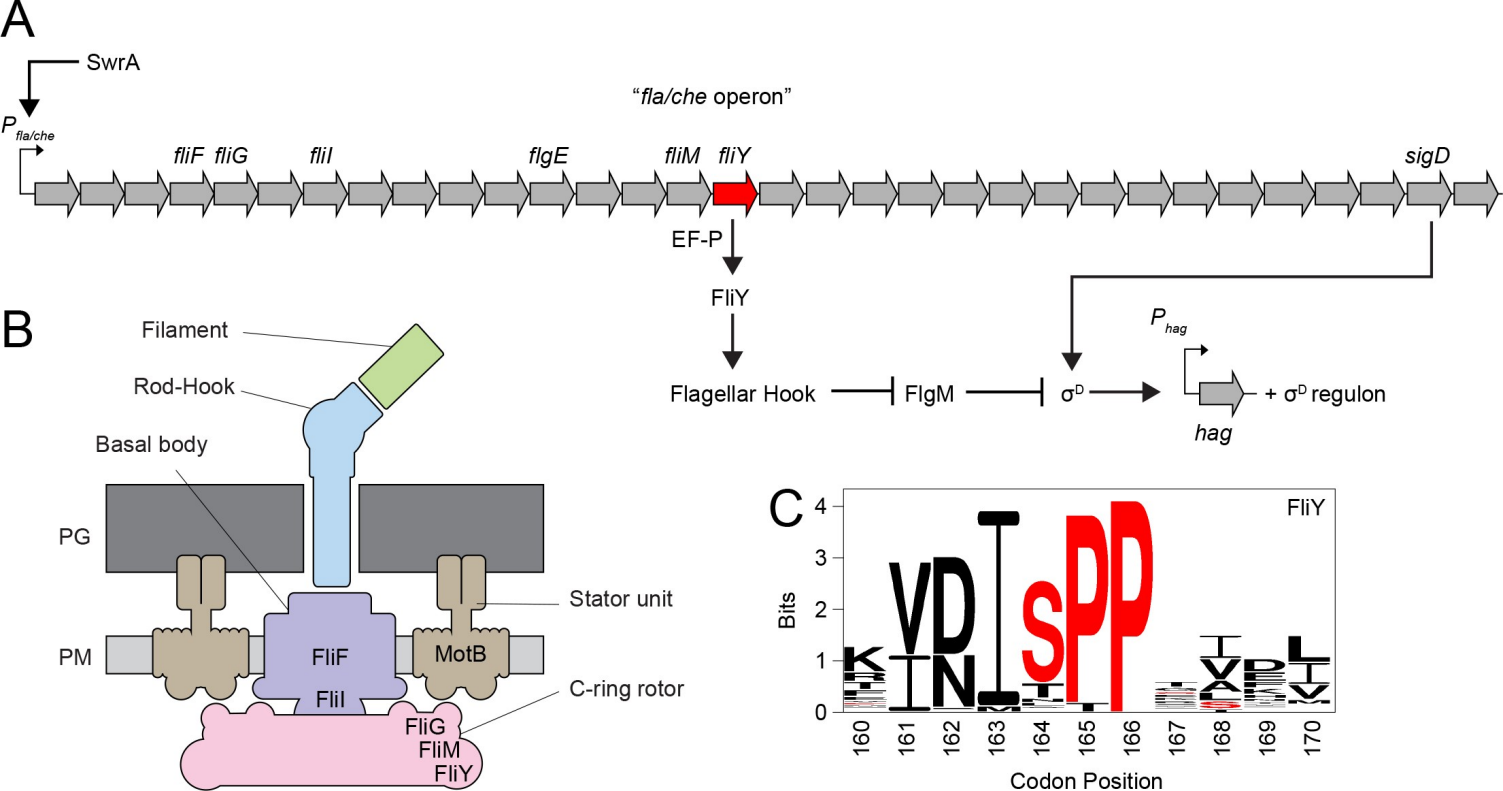
Flagellar genetic hierarchy and structure in *Bacillus subtilis*. Panel A) Schematic depicting the genetic hierarchy of *B*. *subtilis* flagellar biosynthesis. Open arrows represent genes and the position of *fliY* is indicated in red. Bent arrows indicate promoters. Closed arrows indicate activation and T bars indicate inhibition. Panel B) Schematic depicting the putative structure of the *B*. *subtilis* flagellum. The predicted locations of relevant flagellar components are labeled. PG indicates peptidoglycan and PM indicates plasma membrane. Panel C) Sequence logo of 282 FliY homologs. The accession numbers that were included can be found in S1 Table.

**Fig 2:**
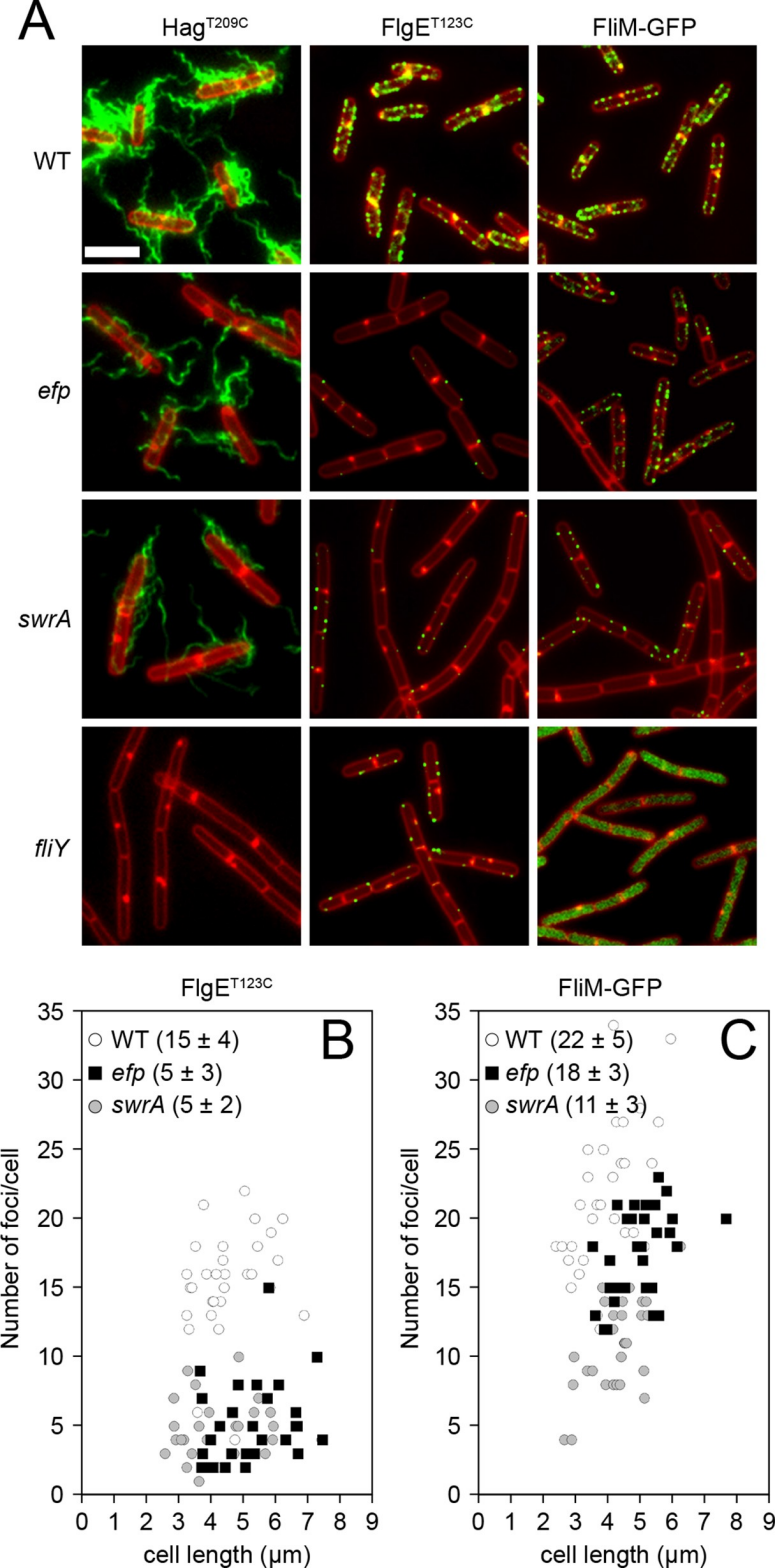
EF-P is required for flagellar hook assembly. Panel A) Fluorescence micrographs of the flagellar filament (Hag^T209C^), hook (FlgE^T123C^), or basal body (FliM-GFP) in the indicated genetic background. Membranes were stained with FM4-64 and false colored red. Filaments and hooks were stained with Alexa Fluor 488 C5 maleimide and false colored green. FliM-GFP was false colored green. Scale bar corresponds to 5 μm. The following strains were used to generate this panel: WT *hag*^*T209C*^ (DS8521), *efp hag*^*T209C*^ (DK1053), *swrA hag*^*T209C*^ (DS8600), *fliY hag*^*T209C*^ (DK2155), WT *flgE*^*T123C*^ (DS7673), *efp flgE*^*T123C*^ (DK1054), *swrA flgE*^*T123C*^ (DK480), *fliY flgE*^*T123C*^ (DK1563), WT *fliM-GFP* (DS1919), *efp fliM-GFP* (DK1055), *swrA fliM-GFP* (DS9515), and *fliY fliM-GFP* (DS5628). Panels B-C) 3-D SIM microscopy and Imaris software were used to quantify the number of basal body puncta (panel B) or hook puncta (panel C) per cell relative to cell length on 30 individual cells per strain. The averages and standard deviations for each strain are provided in the panel legends and the raw values can be found in S2 Table. The following strains were used to generate panel B: WT (DS8521), *efp* (DK1053), and *swrA* (DS8600). The following strains were used to generate panel C: WT (DS7673), *efp* (DK1054), and *swrA* (DK480).

The reason that EF-P is required for swarming motility is unknown. Cells mutated for the master activator of flagellar biosynthesis SwrA lack swarming motility due to reduced transcription of the *fla/che* flagellar operon and a proportional reduction in flagellar number (Fig 1A) [31,33]. To determine if cells mutated for *efp* also had reduced flagellar number, a functional variant of the filament protein Hag that could be labeled with a fluorescent dye (*hag*^*T209C*^) was introduced into various genetic backgrounds [34]. Qualitatively, the *efp* mutant appeared to have fewer filaments than wild type and more closely resembled cells mutated for the master activator of flagellar biosynthesis SwrA (Fig 2A). As swarming motility requires an elevated number of flagella per cell, we infer that the decrease in flagellar number likely accounts for the swarming defect observed upon mutation of *efp*.

Flagella are assembled in a stepwise fashion, with the basal body assembled first, followed by the rod-hook, and finally the filament. Thus, a decrease in flagellar filament number could result from a decrease in either the number of hooks or basal bodies. To determine if mutation of *efp* affected hook and/or basal body number, functional variants of the hook protein FlgE (FlgE^T123C^) or basal body C-ring subunit FliM (FliM-GFP) were introduced into various genetic backgrounds, and fluorescent puncta were quantified with 3D structured illumination microscopy [31,35]. Mutation of *efp* resulted in a decrease in hook number compared to wild type, but the number of basal bodies remained the same (Fig 2A–2C). By contrast and consistent with previous reports, mutation of *swrA* resulted in a decrease in both basal body and hook numbers [24,31] (Fig 2A–2C). We conclude that EF-P is required for completion of a step in flagellar assembly between incorporation of FliM into the C-ring and completion of the hook.

**Fig 3:**
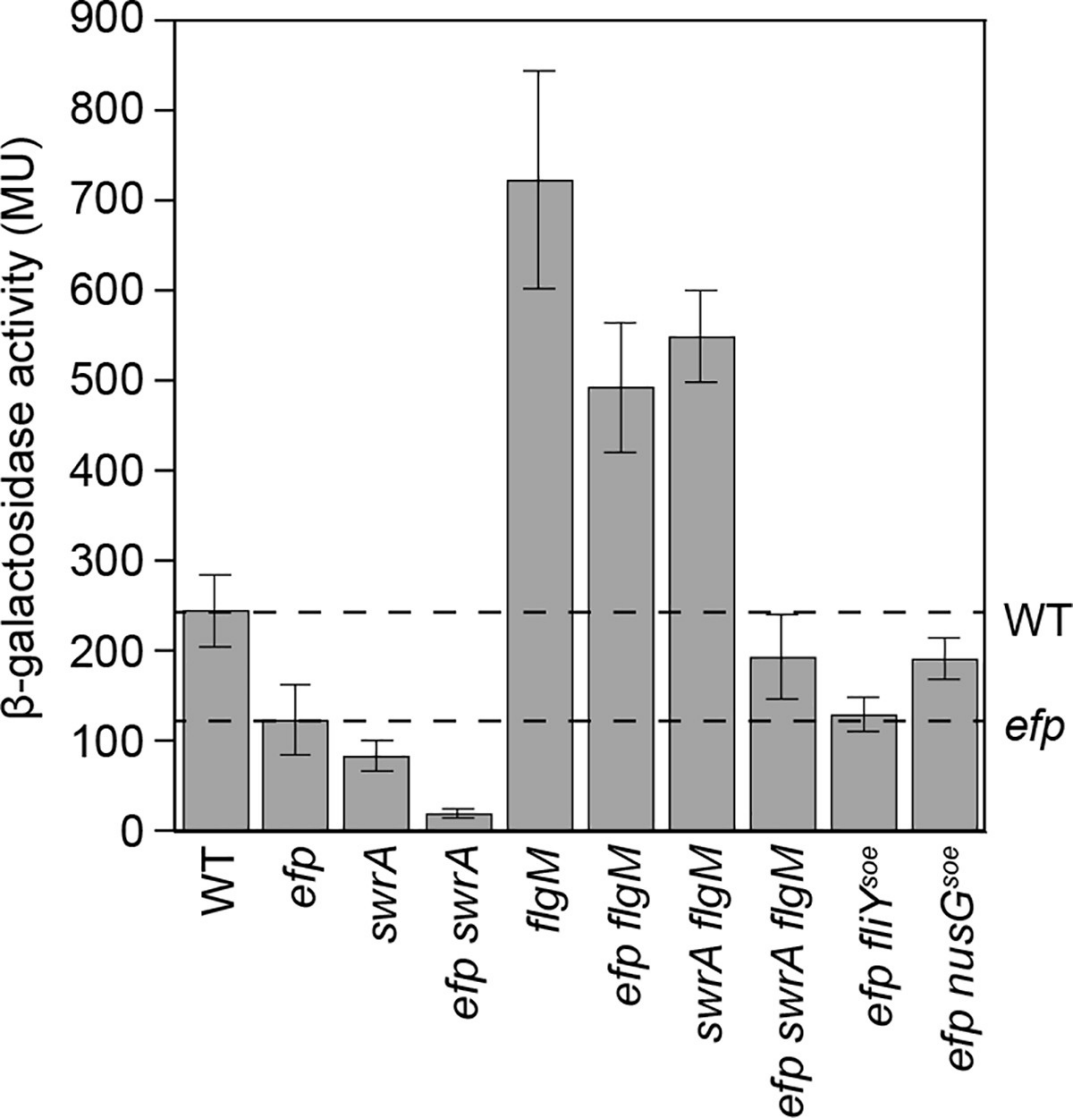
EF-P is required for maximal *P*_*hag*_ expression. β-galactosidase activity reported in Miller Units (MU) of a transcriptional fusion of *lacZ* to the σ^D^-dependent flagellin promoter *P*_*hag*_. The levels of expression in WT and the *efp* mutant are indicated by dashed lines for comparison. Data represent the average of three biological replicates, error bars indicate the standard deviation, and the raw values can be found in S2 Table. The following strains were used to generate this panel: WT (DK5457), *efp* (DK5458), *swrA* (DK7032), *efp swrA* (DK7033), *flgM* (DK7074), *efp flgM* (DK7078), *swrA flgM* (DK7076), and *efp swrA flgM* (DK7077), *efp fliY*^*soe*^ (DK7049), and *efp nusG*^*soe*^ (DK7050).

A defect in hook completion prevents export of the anti-sigma factor FlgM, resulting in a decrease in expression directed by RNA polymerase and the sigma factor σ^D^ (Fig 1A) [30,36]. To determine whether the *efp* mutant was defective in σ^D^-dependent gene expression, a σ^D^-dependent transcriptional reporter in which the promoter of flagellin *P*_*hag*_ fused to β-galactosidase (*P*_*hag*_-*lacZ*) was inserted at an ectopic location in various genetic backgrounds [33,37]. Cells mutated for either *swrA* or *efp* showed a decrease in expression from the *P*_*hag*_ promoter, and expression was partially restored to either *swrA* or *efp* mutants by mutation of *flgM* (Fig 3). SwrA and EF-P appeared to enhance *P*_*hag*_*-lacZ* expression by different pathways, however, as an *efp swrA* double mutant synergized to decrease promoter activity (Fig 3). Further, expression in an *efp swrA flgM* triple mutant remained low relative to either the *swrA flgM* or the *efp flgM* double mutants (Fig 3). We conclude that EF-P promotes hook completion and σ^D^-dependent gene expression by a mechanism unrelated to SwrA activation of the *P*_*fla/che*_ promoter.

### EF-P alleviates pausing at XPPX motifs

**Fig 4:**
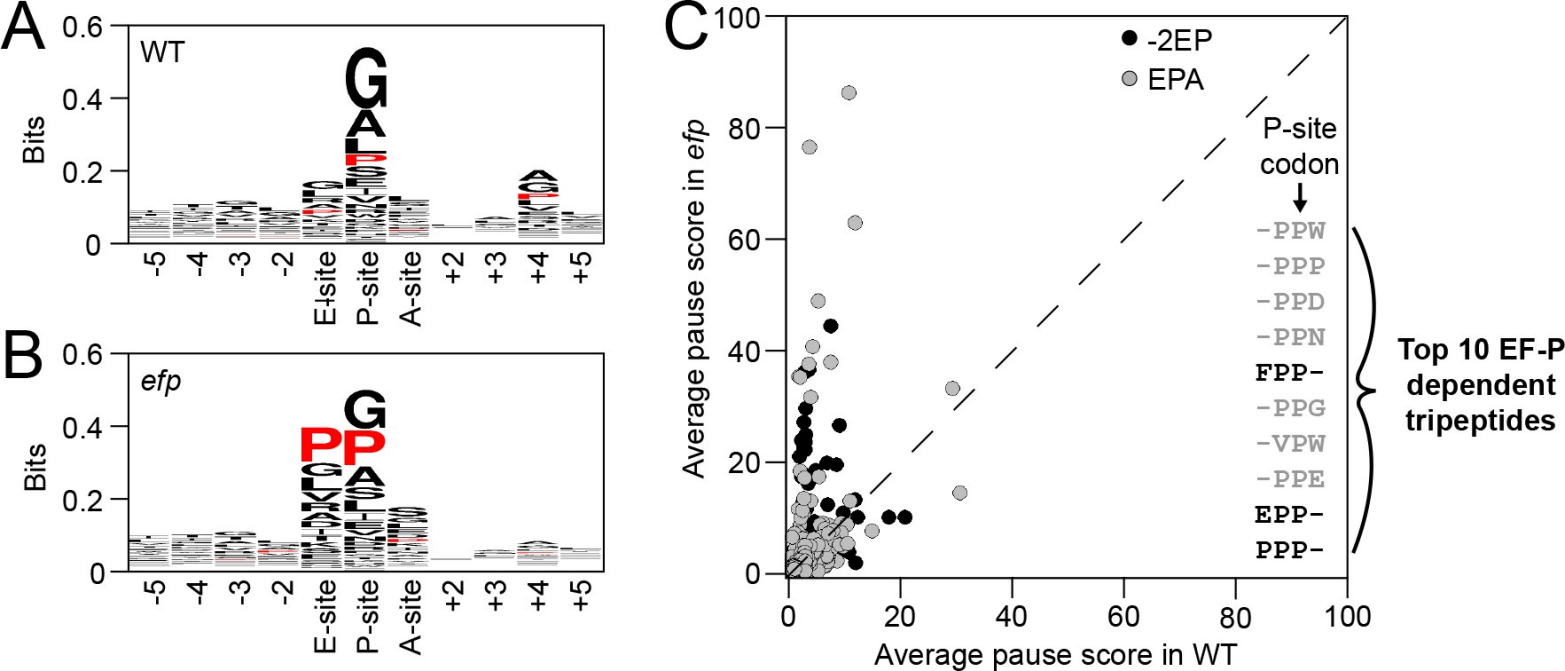
EF-P alleviates ribosome pausing at XPPX motifs. Panels A-B) Weighted sequence logos of amino acid sequences in which the P-site codon had a pause score of 10 or greater in the ribosome profiling datasets from WT (DK1042) or *efp* (DK2050). Panel C) Average pause scores of tripeptides centered around the P-site (EPA, grey circles) or E site (-2EP, black circles) of the ribosome in WT (DK1042) and *efp* mutant (DK2050). In both cases, only the pause score for the P-site codon was used to determine the average. The dashed line indicates the distribution expected for equal scores in both strains. The 10 tripeptides with the highest pause score in the *efp* mutant are listed and the average pause scores for all 8,000 tripeptides at both positions can be found in S5 Table.

One way that EF-P could promote flagellar biosynthesis is if it alleviated ribosome pausing as it does in a number of other organisms [1–3]. To determine whether EF-P in *B*. *subtilis* alleviates ribosome pausing, the ribosome pause sites of wild type and an *efp* mutant were compared. In brief, mRNA fragments protected by ribosome footprinting were purified, subjected to Illumina sequencing, and the codons in the ribosomal P-site were identified using the 3’ end assignment method [10]. Each codon in the genome was then assigned a pause score, defined as the number of reads that mapped to a particular position divided by the average read density for the corresponding gene (S3 Table). Approximately 250 codons in 180 genes had a pause score that was at least 10-fold higher in the *efp* mutant compared to WT (S4 Table). In the absence of EF-P, proline codons were enriched in both the ribosome P-site and E-site, and the tripeptides encompassing the “-2”, “E”, “P”, and “A” sites showed an enrichment of pausing at PPX and XPP motifs (Fig 4A–4C, S5 Table). We conclude that in the absence of EF-P, *B*. *subtilis* ribosomes paused more frequently at XPPX motifs, consistent with that reported in other organisms.

The 180 genes that experienced increased ribosome pausing in the absence of EF-P were predicted to be diverse in function (S6 Table). Five genes with EF-P-alleviated pause sites appeared to be directly related to the flagellum: *fliI* (encoding the secretion accessory protein FliI), *fliF* (encoding the basal body base plate FliF), *motB* (encoding the stator component MotB), *sigD* (encoding the alternative sigma factor σ^D^), and *fliY* (encoding the C-ring component FliY), (Fig 1B, S4 Table) [38]. FliF, σ^D^, and FliY are all required for hook completion and their translational impairment might be consistent with the *efp* mutant phenotype but it wasn’t clear which, or how many, of the sites were directly responsible [24,35]. Moreover, EF-P-alleviated pause sites were observed in several essential genes, even though mutation of *efp* did not substantially reduce growth rate (S4 Table) [12,39]. Thus, as ribosome pauses were found in at least 5 genes known to be involved in motility (not including genes of unknown function), and the location of EF-P-alleviated translational pausing was not necessarily predictive of phenotype, we concluded that ribosome profiling alone was insufficient to identify the hook-promoting EF-P target.

### EF-P can be suppressed by mutations in ribosome-associated proteins

**Fig 5:**
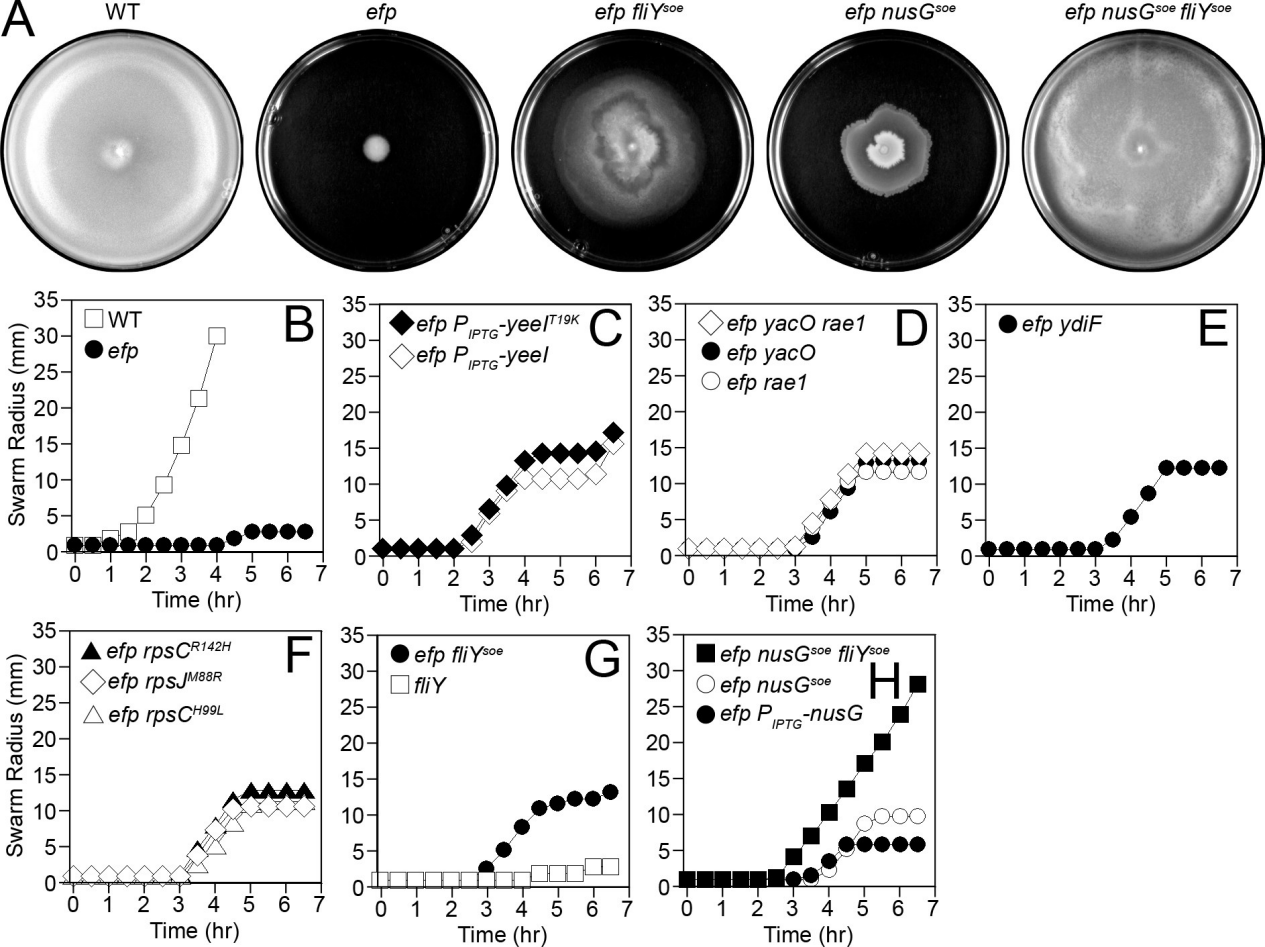
The *efp* mutant swarming defect can be genetically suppressed. Panel A) Top views of centrally inoculated swarm plates incubated overnight at 37°C and imaged against a black background. Zones of colonization appear light grey. The following strains were used to generate the panel: WT (DK1042), *efp* (DK2050), *efp fliY*^*soe*^ (DK5518), *efp nusG*^*soe*^ (DK5955), and *efp nusG*^*soe*^ *fliY*^*soe*^ (DK6640). Panels B-H) Quantitative swarm expansion assays in which mid-log phase cultures were concentrated and used to inoculate swarm agar plates. Swarm expansion was monitored along the same axis every 30 min for 6.5 hrs. Each data point represents the average of three replicates and the raw values can be found in S2 Table. The following strains were used as the inoculum: B) WT (DK1042) and *efp* (DK2050). C) efp *P*_*IPTG*_-*yeeI*^*T19K*^ (DK2779) and *efp P*_*IPTG*_-*yeeI* (DK2777). D) *efp yacO rae1* (DK5415), *efp yacO* (DK5413), and *efp rae1* (DK5414). E) *efp ydiF* (DK4093). F) *efp rpsC*^*R142H*^ (DK6656), *efp rpsJ*^*M88R*^ (DK6657), and *efp rpsC*^*H99L*^ (DK6656). G) *efp fliY*^*soe*^ (DK5518) and *fliY* (DK1481). H) *efp nusG*^*soe*^ *fliY*^*soe*^ (DK6640), *efp nusG*^*soe*^ (DK5955), and *efp P*_*IPTG*_-*nusG* (DK5412).

As an alternative approach to determine how EF-P increased hook number, spontaneous suppressors were isolated that restored swarming motility to an *efp* mutant. Cells mutated for *efp* were initially incapable of migrating from the site of inoculation on swarming motility agar (Fig 5A and 5B), but after prolonged incubation, second-site mutations that suppressed the need for EF-P emerged from the central colony as motile flares. Twenty-four suppressors of *e**fp* (*soe*) mutations were independently isolated and each suppressor resulted in a partial restoration of swarming motility. The location of each suppressor mutation in the genome was identified using a combination of SPP1-mediated transduction linkage mapping and whole genome sequencing. The mutations were organized into 6 different classes based on their chromosomal location (Table 1).

**Table 1:** Suppressors of *efp* (*soe*).

Suppressor	Genotype^1^
**Class I- mutations in *yeeI***	
***soe2***	*P*_*yeeI*_ TCGAAA > TTGAAA (-35 element)
***soe5***	*P*_*yeeI*_ TCGAAA > TTGAAA (-35 element)
***soe10***	*P*_*yeeI*_ TCGAAA > TTGAAA (-35 element)
***soe19***	*P*_*yeeI*_ TCGAAA > TTGAAA (-35 element)
***soe21***	*P*_*yeeI*_ TCGAAA > TTGAAA (-35 element)
***soe28***	*P*_*yeeI*_ TCGAAA > TTGAAA (-35 element)
***soe29***	ACG > AAG (YeeI^T19K^)
**Class II- mutations in the *yacO/rae1* operon**	
***Subclass A- Mutations in yacO***	
***soe11***	Frameshift at nucleotides 274–281 A^7^ > A^8^
***soe15***	Frameshift at nucleotides 274–281 A^7^ > A^6^
***soe20***	Frameshift at nucleotides 274–281 A^7^ > A^6^
***soe26***	Deletion of nucleotides 609–613
***Subclass B- Mutations in rae1***	
***soe7***	Frameshift at nucleotides 183–189 A^6^ > A^7^
***soe9***	CGG > CCG (Rae1^R166P^)
***soe13***	Deletion of nucleotides 469–473
**Class III- mutations in *ydiF***	
***soe12***	Duplication of nucleotides 1073–1077
***soe16***	Duplication of nucleotides 814–873
***soe18***	AAA > GAA (YdiF^K366E^)
***soe22***	AAC > CAC (YdiF^N464H^)
***soe23***	TAC > TAA (YdiF^Y246STOP^)
**Class IV- mutations in ribosomal proteins**	
***Subclass A- Mutations in S3***	
***soe1***	CGT > CAT (S3^R142H^)*
***soe3***	CAC > CTC (S3^H99L^)^†^
***Subclass B—Mutations in S10***	
***soe24***	ATG > AGG (S10^M88R^)
**Class V- mutations in *fliY***	
***soe8***	TCA > GCA (FliY^S164A^)
**Class VI–mutations in *nusG***	
***soe17***	AAC > AGC (NusG^N21S^)

Suppressor of *efp* (*soe*) genoytpes.

^1^The wild type sequence is listed prior to the greater than sign, which is followed by the mutant sequence. No additional mutations were identified unless otherwise indicated.

*Mutations at genome position 26,502 (C > T) between *tadA* and *dnaX* and position 302,890 (A > G) between *iscS* and *braB* were also detected.

^†^An additional missense mutation encoding NrdI^K74E^ (AAG > GAG) was also detected

#### Class I *soes*–increased expression of YeeI

Class I mutations were identified by transposon-linked SPP1 generalized phage transduction mapping and subsequent candidate gene sequencing. The seven class I *soes* were located in the *yeeI* locus, which encodes YeeI, a conserved protein of unknown function that has been connected to either transcription and/or translation in Bacteria and Eukaryotes, respectively (Table 1; S1A Fig, S2 Fig) [40,41]. Six class I *soes* introduced identical mutations in a putative -35 region of a potential σ^A^-dependent promoter (*P*_*yeeI*_*)*, mutating it closer to the σ^A^ consensus sequence (Table 1, S1A Fig). β-galactosidase activity was observed when wild type *P*_*yeeI*_ was cloned upstream of *lacZ* and when the *soe* mutation was incorporated in the reporter construct, the expression increased (S3 Fig). To test if increasing expression of *yeeI* was sufficient to suppress *efp*, the *yeeI* open reading frame was cloned under the control of an IPTG-inducible promoter, introduced into the *efp* mutant background, and swarming motility was monitored in the presence of IPTG. While the *efp* mutant was incapable of moving from the site of inoculation, *efp P*_*IPTG*_*-yeeI* was able to swarm at a rate similar to the class I suppressors (Fig 5C). The final class I *soe* was a missense mutation in the *yeeI* open reading frame (YeeI^T19K^) (Table 1, S1A Fig). We infer that the *yeeI*^*T19K*^ allele acts to similarly increase YeeI levels as no additive effect on swarming motility was observed when *yeeI*^*T19K*^ was overexpressed from an IPTG-inducible promoter (Fig 5C). We conclude that class I *soes* suppress *efp* by increasing expression of YeeI.

#### Class II *soes*–loss-of-function mutations in *yacO* and *rae1*

Class II mutants were identified by Illumina whole genome sequencing and subsequent sequencing of candidate genes. All seven class II *soes* fell within two adjacent co-transcribed genes that encode the conserved 23S RNA methyltransferase homolog YacO and the ribosome-associated endonuclease Rae1 (Table 1, S1B Fig; S2 Fig) [42]. Most of the mutations introduced frameshifts to one or the other of the open reading frames, but one *soe* introduced a missense mutation in Rae1^R166P^ (Table 1). Consistent with class II *soes* resulting in loss-of-function of either YacO or Rae1, insertion/deletion of either open reading frame was sufficient to suppress *efp* (Fig 5D). Further, mutation of either gene appeared to affect the same pathway, as double mutation of *yacO* and *rae1* was not additive and phenocopied either single mutant alone (Fig 5D). We conclude that class II *soes* suppress *efp* by disrupting YacO or Rae1 activity.

#### Class III *soes*–loss-of-function mutations in *ydiF*

Class III mutants were identified by Illumina whole genome sequencing and subsequent sequencing of candidate genes. The five class III *soes* were located in the *ydiF* locus, encoding YdiF, a conserved putative ATP binding protein that resembles ribosome associated ATPases (Table 1, S1C Fig; S2 Fig) [43]. Each class III mutation likely conferred a loss-of-function in YdiF as two introduced frameshift mutations, one introduced a nonsense mutation, and the final two introduced missense mutations (YdiF^K366E^, YdiF^N464H^) into the *ydiF* open reading frame (Table 1). Consistent with all class III *soes* resulting in loss-of-function of *ydiF*, insertion/deletion mutation of *ydiF* was sufficient to suppress *efp* (Fig 5E). We conclude that class III *soes* suppress *efp* by disrupting YdiF activity.

#### Class IV *soes*–mutations in ribosomal proteins

Class IV mutants were identified by Illumina whole genome sequencing. The three class IV *soes* were missense mutations within the genes *rpsC* and *rpsJ* that encode the conserved ribosome protein subunits S3 and S10, respectively (Table 1, Fig 5F; S2 Fig). In the case of the missense mutation in *rpsJ* (S10^M88R^), whole genome sequencing showed no other mutation in the chromosome. Both *rpsC* mutants however, had additional unlinked mutations (Table 1). Attempts to directly establish linkage between the *rpsC* and *rpsJ* alleles and suppression of the *efp* motility phenotype were unsuccessful as the complex arrangement of other essential genes in the long operon did not permit insertion of a plasmid for loop-in-loop-out allelic replacement. Despite an inability to conclusively demonstrate linkage, we nonetheless infer that the mutations in *rpsJ* and *rpsC* confer an altered function as both gene products are essential [44–46]. Ribosome protein S3 is an RNA helicase thought to unwind the mRNA transcript, while protein S10 interacts with the P-site tRNA as well as the transcriptional anti-termination factor NusG [47–50]. In sum, suppressor classes I-IV support a model in which EF-P promotes swarming motility either through protein translation or through some other ribosome-associated function.

### EF-P enhances translation of FliY

#### Class V *soe*–mutation in *fliY*

The one class V mutant was identified by Illumina whole genome sequencing and was found to contain a missense mutation in the *fliY* locus (*fliY*^*S164A*^), encoding the flagellar C-ring component FliY within the *fla/che* operon (Table 1, Fig 5G) [51–53]. FliY could be the target of EF-P activity that accounts for the swarming motility defect in the *efp* mutant background as FliY is a structural component of flagella and the mutated Ser164 immediately precedes two consecutive proline residues of an SPP motif (Fig 1B and 1C). Consistent with being a potential target, mutation of FliY abolished swarming motility and reduced flagellar hook numbers similar to that observed in cells mutated for *efp* (Fig 5G, Fig 2A). Whereas cells mutated for *efp* had abundant FliM-GFP puncta, cells mutated for *fliY* had few to none (Fig 2A). We infer that FliY is essential for stabilizing the FliM-GFP reporter construct at flagellar basal bodies, and that the lack of EF-P may reduce but not abolish FliY expression. We predict that the phenotype of the *efp* mutant may, at least in part, be the result of a reduction in FliY protein levels.

**Fig 6:**
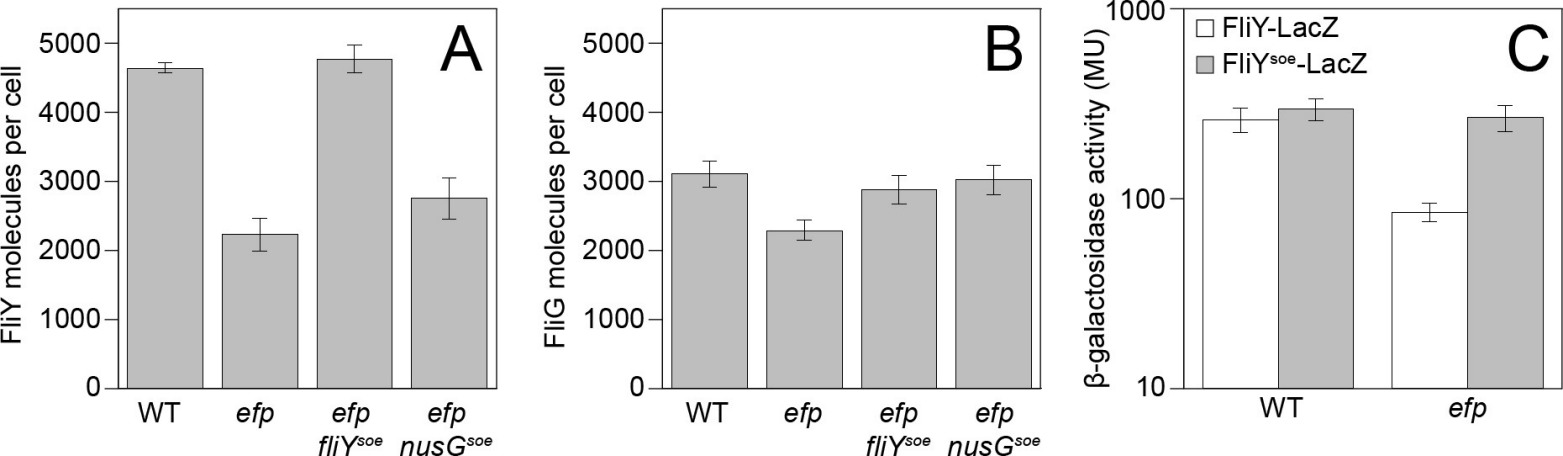
EF-P enhances the translation of FliY. Panels A-B) Quantitative Western blot analysis of FliY and FliG. Error bars indicate the standard deviation of 3 biological replicates and the raw values can be found in S2 Table. The following strains were used to generate these panels: WT (DK1042), *efp* (DK2050), *efp fliY*^*soe*^ (DK5518), and *efp nusG*^*soe*^ (DK5955). Panel C) β-galactosidase activity reported in Miller Units (MU) of a translational fusion of *lacZ* to *fliY*. Error bars indicate the standard deviation of 3 biological replicates and the raw values can be found in S2 Table. The following strains were used to generate this panel: WT *fliY-lacZ* (DK5185), *efp fliY-lacZ* (DK5186), WT *fliY*^*soe*^*-lacZ* (DK5168), and *efp fliY*^*soe*^*-lacZ* (DK5169).

To determine whether FliY levels were reduced in the absence of EF-P, quantitative Western blot analysis was performed on lysates of exponentially-grown *B*. *subtilis* using an antibody raised against the FliY protein and an infra-red based detection system. A standard curve was generated with purified FliY protein that was diluted with a cell lysate of a *fliY* mutant to maximize transfer similarity to the whole cell lysates with which it would be compared. Cells mutated for *efp* had a fifty percent reduction in FliY proteins per cell whereas the number of another C-ring protein, FliG, was only slightly diminished, despite the fact that both genes are encoded in the same operon (Fig 1A, Fig 6A and 6B). Moreover, the *fliY*^*soe*^ allele increased the number of FliY molecules in the absence of EF-P to a number similar to wild type (Fig 6A). We conclude that there is a specific reduction of FliY protein levels in the absence of EF-P. We infer that in the absence of EF-P, the shortage of FliY results in a flagellar subpopulation with incomplete C-rings that fail to become active for early-class secretion and fail to synthesize hooks.

**Fig 7:**
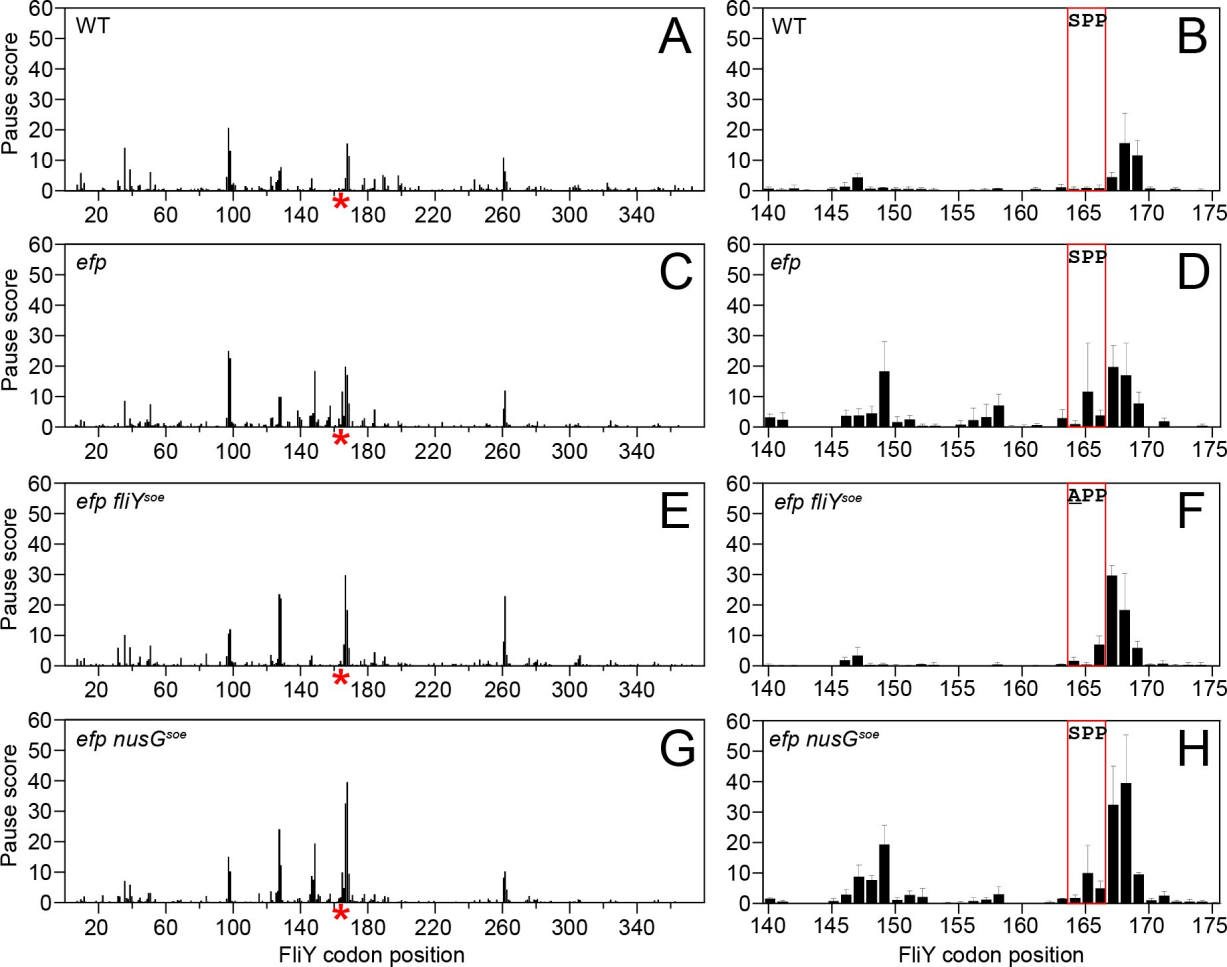
EF-P alleviates ribosome pausing at FliY^SPP^. Panels A, C, E, G) Average ribosome profiling pause scores of each codon within the FliY open reading frame. The position of the SPP motif is indicated by a red asterisk on the X-axis. Panels B, D, F, H) Average pause scores for FliY codons 145–175. The box indicates the location of the SPP motif. Error bars indicate the standard deviation of 3 biological replicates. The following strains were used to generate this figure: WT (DK1042), *efp* (DK2050), *efp fliY*^*soe*^ (DK5518), and *efp nusG*^*soe*^ (DK5955).

One way in which EF-P might increase FliY abundance is by potentiating translation of the *fliY* transcript. In wild type ribosome profiling, a substantial pause site was observed at FliY codons 167–169 (Fig 7A and 7B). In the *efp* mutant, however, this peak was expanded to include the SPP motif codons 165–166 (Fig 7C and 7D). Further, two additional peaks appeared centered on codons 146–151 and 155–158, perhaps consistent with ribosome accumulation upstream of the SPP motif due to ribosome queuing [10] (Fig 7C and 7D). The *fliY*^*soe*^ suppressor mutation not only reduced ribosome pausing at proline codon 165 but also eliminated the upstream peaks attributed to ribosome accumulation (Fig 7E and 7F). Finally, mutation of *efp* reduced expression from a FliY translational fusion to LacZ and expression was increased to wild type levels when the reporter also encoded the *fliY*^*soe*^ mutation (Fig 6C). The *fliY*^*soe*^ suppressor allele did not, however, increase expression of the *P*_*hag*_*-lacZ* transcriptional reporter in the *efp* mutant (Fig 3). We conclude that EF-P specifically reduces ribosome pausing at FliY proline 165 to increase FliY translation to levels necessary to partially support swarming, but that EF-P may also promote swarming by additional mechanisms.

### EF-P promotes σ^D^ activity independent of FliY

#### Class VI *soe*–mutation in *nusG*

The one class VI mutant was identified by Illumina whole genome sequencing and was found to contain a missense mutation in the *nusG* locus (*nusG*^*N21S*^), encoding the conserved transcription elongation factor NusG (Table 1, S2 Fig) [54,55]. Linkage was confirmed when the *nusG*^*soe*^ allele was independently generated by allelic replacement and demonstrated to restore swarming to the *efp* mutant (Fig 5H). *nusG*^*soe*^ is likely not a loss-of-function mutation as an insertion/deletion mutation of *nusG* did not rescue swarming in the absence of EF-P (S1D Fig). Instead, *nusG*^*soe*^ may be a gain-of-function mutation, as overexpression of *nusG* from an IPTG-inducible promoter was also sufficient to partially rescue swarming motility to the *efp* mutant (Fig 5H). We conclude that the class VI suppressor causes either a gain or a change of function in NusG.

To determine whether NusG^soe^ suppressed the absence of EF-P in a manner similar to class V *soe* mutations, quantitative Western blot analysis was conducted using an anti-FliY antibody. Unlike the FliY^soe^ allele, NusG^soe^ did not substantially increase FliY protein levels (Fig 6A). Moreover, ribosome profiling indicated that pausing was similar across the genome in both the *efp* and *efp nusG*^*soe*^ mutant, and the specific pattern of pausing at FliY^SPP164-166^ was unaltered (Fig 7G and 7H). Instead, *nusG*^*soe*^ increased *P*_*hag*_*-lacZ* expression compared to the *efp* mutant alone (Fig 3). We conclude that the *nusG*^*soe*^ allele does not rescue swarming motility in the absence of EF-P by increasing FliY levels, and suppression was instead correlated with an increase in *P*_*hag*_ expression.

**Fig 8:**
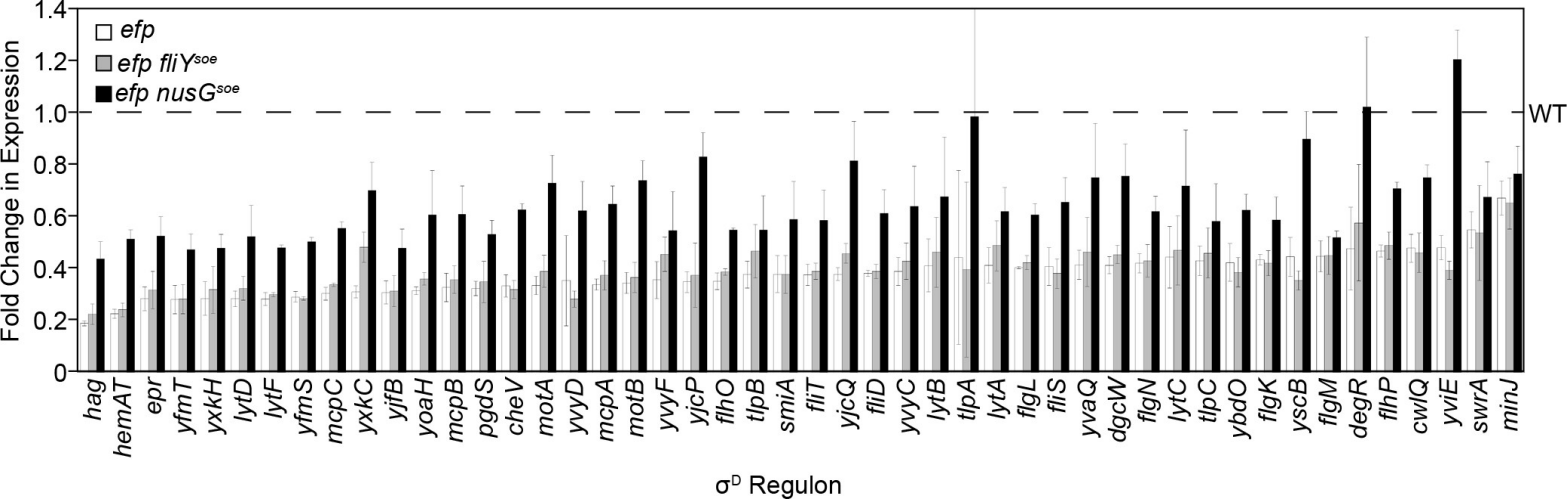
σ^D^-dependent gene expression decreases in the absence of EF-P. Fold change in expression detected by RNA sequencing of genes in the σ^D^ regulon relative to wild type. The levels of expression in wild type are indicated by a dashed line. Error bars represent the standard deviation of 3 biological replicates. The following strains were used to generate this figure: WT (DK1042), *efp* (DK2050), *efp fliY*^*soe*^ (DK5518), and *efp nusG*^*soe*^ (DK5955).

*P*_*hag*_ is a member of the σ^D^ regulon and to determine whether the *P*_*hag*_ expression defect in the *efp* mutant was specific to that promoter, RNA sequencing analysis was performed on the same lysates used for ribosome profiling. Comparative transcriptomics indicated that there was a global decrease in expression of the σ^D^ regulon in the *efp* mutant compared to wild type (Fig 8, S8 Table) [33,56,57]. Introduction of the *nusG*^*soe*^ allele, but not the *fliY*^*soe*^ allele, to the *efp* mutant background increased expression of the majority of σ^D^-dependent genes (Fig 8, S8 Table). An increase in σ^D^-dependent gene expression was not sufficient to rescue swarming to the *efp* mutant however, as *efp* mutants that lacked FlgM and/or overexpressed σ^D^ did not swarm (S1E Fig). Thus, we infer that the increase in σ^D^-dependent gene expression in the *nusG*^*soe*^ allele is an indirect consequence of the mechanism of swarming suppression. Whatever the mechanism of *nusG*^*soe*^ suppression, it appears to be in a pathway parallel to that of *fliY*^*soe*^ as an *efp* mutant expressing both alleles simultaneously exhibited swarming motility that was greater than either alone (Fig 5A and 5H). We conclude that EF-P promotes swarming by enhancing FliY translation and by a second, NusG-mediated mechanism.

## Discussion

Translation elongation factor P (EF-P) is conserved in all domains of life and has been shown to alleviate ribosome pausing at a subset of sequences encoding tandem proline residues (XPPX motifs) [8,10,58]. In many organisms, EF-P is required for growth, presumably because it enhances translation of one or more XPPX-containing essential proteins, the ValS aminoacyl-tRNA synthetase in particular [4–6,59]. In *B*. *subtilis* however, *efp* mutants have negligible growth defects and instead are specifically incapable of a flagellar-mediated surface motility called swarming [12,13,39]. We show here that *B*. *subtilis* EF-P alleviates ribosome pausing at XPPX motifs in a manner nearly indistinguishable from other organisms. We further attribute the *efp* mutant swarming defect to a decrease in flagellar number at the level of flagellar hook biosynthesis, and we analyze spontaneous suppressor mutants that restore swarming motility. Many of the suppressors were in ribosome subunits or ribosome-associated factors and were likely compensatory. One suppressor mutant, however, was in the motility target of EF-P and changed a ribosome pause-inducing SPP motif to APP in the flagellar C-ring component, FliY.

FliY is homologous to the protein FliN found in the flagellar C-ring of other bacteria, and the *efp* mutant flagellar assembly defect is consistent with FliY being a motility-related EF-P target [24,60]. A *fliY* deletion does not perfectly phenocopy mutation of *efp*, as FliY is necessary for flagellar C-ring assembly and all forms of flagellar motility, whereas the *efp* mutant has wild type basal body numbers and can swim but not swarm (Fig 2A) [13]. Moreover, the *fliY* mutant lacks flagellar filaments whereas the *efp* mutant does not, perhaps because FliY, like FliN, may be a docking point for the late class flagellar secretion protein FliH (Fig 2) [61,62]. The absence of EF-P instead increases ribosome pausing and decreases FliY copy number, thereby reducing the frequency of flagella that complete basal body assembly and activate early-class type III secretion [24].

Why EF-P is needed to specifically relieve translational pausing of FliY is unclear. The need for EF-P may be unavoidable as the SPP motif falls within a highly conserved sequence of residues that are nearly invariant. The FliY^soe^ allele in otherwise wild type cells, however, exhibited nearly wild type levels of swarming motility suggesting that an EF-P-independent variant is indeed tolerated (Fig 1C, S1F Fig). Alternatively, EF-P pausing relief may play a regulatory role. While EF-P in *B*. *subtilis* is constitutively expressed, it is post-translationally modified by 5-aminopentanolylation which is predicted to be built through the sequential maturation of at least 3 EF-P modification intermediates [63]. Moreover, previous work has shown that the modification state of *B*. *subtilis* EF-P alters its activity and therefore may represent a method of regulating EF-P function [39,63]. We note that while FliN of *E*. *coli* does not encode an XPPX motif, translational pausing in the absence of EF-P is nonetheless conserved at a series of four consecutive valine residues, perhaps indirectly due to increased ribosome pausing in ValS and a concomitant decrease in tRNAs charged with valine (S4 Fig) [10].

Suppression of the *efp* mutant swarming defect could be achieved through mutation of 7 additional loci, many of which are broadly conserved and could be readily related to the translational machinery (S2 Fig, S7 Table). The location of these additional suppressors may provide insight into the mechanism by which EF-P promotes translation in diverse organisms. Homologs of YeeI are highly conserved and poorly studied, but one YeeI homolog in humans, TACO1, has been implicated in activating the translation of Cox1, which contains 4 XPPX motifs [41]. YdiF is a broadly conserved member of the ABC-F family of ATPases which comprises many proteins known to interact with the ribosome such as EF-3 in Eukaryotes and EttA in *E*. *coli* [43]. YacO is homologous to RlmB in *E*. *coli*, a highly conserved protein that methylates the 23S rRNA guanosine G2251 within the ribosomal peptidyltransferase domain [64]. Rae1 has been recently shown to act as a ribosomal A-site endoribonuclease, and it was hypothesized that ribosome stalling may increase its access to its mRNA substrate and thereby increase its activity [42]. Finally, S3 and S10 are components of the small subunit of the ribosome itself: S3 is involved in mRNA processivity and S10 is involved in binding to the P-site tRNA [48,50]. Further, the residue altered by *soe24* (S10^M88R^) has been implicated in the direct interaction with the last protein identified by *efp* suppressor analysis, NusG [49].

NusG couples transcription and translation in *E*. *coli* by binding both RNA polymerase and the leading ribosome on the transcript to promote transcriptional elongation [47,49]. In *B*. *subtilis*, however, NusG is thought to do the opposite and promote transcriptional pausing [65,66]. In *E*. *coli*, NusG also binds to the ribosome but whether it does so in *B*. *subtilis* and how the *nusG*^*soe*^ allele suppresses the *efp* swarming defect is unclear [47,49]. NusG^soe^ appears to be a gain-of-function allele that does not increase FliY protein levels but rather increases the expression of σ^D^-dependent late-class flagellar genes, including the flagellar filament (Fig 3, Fig 8). The increase in σ^D^ -dependent gene expression, however, is likely an indirect effect of suppression as artificial activation of σ^D^ was insufficient to restore swarming to the *efp* mutant (S1E Fig). While the mechanism by which NusG^N21S^ suppresses the *efp* mutant swarming defect is unknown, it appears to operate in parallel to the alleviation of ribosome pausing in FliY, as the *nusG*^*soe*^ and *fliY*^*soe*^ alleles synergized to enhance swarming in the *efp* mutant background (Fig 5H). The majority of flagellar genes including both *fliY* and *sigD* are encoded on what is thought to be a single transcript from the 27kb 32 gene *fla/che* operon (Fig 1) [67–70]. Perhaps NusG is somehow involved in the expression of long transcripts.

Ultimately, EF-P alleviates ribosome pausing at some but not all XPPX motifs, and the context that causes a particular primary sequence to trigger stalling is unclear. For example, cells fail to swarm when ribosomes pause at an SPP motif in the *fliY* transcript and swarming is restored by substitution to a APP motif, another site that also experiences strong pausing elsewhere in the genome. Moreover, even in situations where ribosome pausing is severe, there may or may not be phenotypic consequences. For example, ribosomes also pause at and accumulate upstream of a PPP motif in the *valS* transcript but little to no growth defect is observed, and unlike the case in *E*. *coli*, pauses at valine residues are not enriched in *B*. *subtilis* (S5 Fig, Compare Fig 3B to S4D Fig). Thus, one cannot predict whether ribosomes pause at particular motifs by bioinformatics, and it may be difficult to predict the phenotypes of *efp* mutants simply from ribosome profiling data sets.

Our work supports previous observations in *E*. *coli* that the phenotypic effect of EF-P may be most significant for pauses in proteins for which relative stoichiometry is important. For example, EF-P-alleviated pausing has been shown to be important for the maintenance of subunit ratio for the F_1_F_0_ ATPase [8,71]. Moreover, EF-P relieves translational pausing within CadC, a transcriptional activator that is antagonized by direct interaction with LysP [3]. Thus translational pausing creates a stoichiometric imbalance and results in constitutive antagonism of CadC and deactivation of the CadC transcriptional target [3]. Here we provide evidence that EF-P supports synthesis of the protein FliY, which when in stoichiometric deficiency limits the cells ability to complete flagellar basal body biosynthesis, increase flagellar number, and perform swarming motility. We broadly speculate that biological systems which depend on stoichiometry may be particularly sensitive to translational pausing and thus display enhanced phenotypic dependency on EF-P.

## Methods

### Strains and growth conditions

*B*. *subtilis* and *E*. *coli* strains were grown in lysogeny broth (LB) (10 g tryptone, 5 g yeast extract, 5 g NaCl per L) or on LB plates fortified with 1.5% Bacto agar at 37°C. When appropriate, antibiotics were included at the following concentrations: 100 μg/ml ampicillin, 10 μg/ml tetracycline, 100 μg/ml spectinomycin, 5 μg/ml chloramphenicol, 5 μg/ml kanamycin, and 1 μg/ml erythromycin plus 25 μg/ml lincomycin (*mls*). Isopropyl β-D-thiogalactopyranoside (IPTG, Sigma) was added to the medium at the indicated concentration when appropriate. Strain construction and suppressor isolation details are described in the S1 Text. Strains used in this study are listed in Table 2, plasmids are listed in S9 Table, and primers are listed in S10 Table.

**Table 2:** Strains.

Strain	Genotype	Reference
DK480	*swrA*::*kan ΔflgE amyE*::*P*_*fla/che*_*-flgE*^*T123C*^ *cat*	[24]
DK1042	*comI*^*Q12L*^	[72]
DK1053	*efp*::*tet ΔfliM amyE*::*P*_*fla/che*_*-fliM-GFP spec*	
DK1054	*efp*::*tet ΔflgE amyE*::*P*_*fla/che*_*-flgE*^*T123C*^ *cat*	
DK1055	*efp*::*tet Δhag amyE*::*P*_*hag*_*-hag*^*T209C*^ *spec*	
DK1481	*comI*^*Q12L*^ *ΔfliY*	
DK1563	*comI*^*Q12L*^ *ΔfliY ΔflgE amyE*::*P*_*fla/che*_*-flgE*^*T123C*^ *cat*	
DK2050	*comI*^*Q12L*^ *Δefp*	[12]
DK2154	*comI*^*Q12L*^ *ΔfliMY*	
DK2155	*comI*^*Q12L*^ *ΔfliMY amyE*::*P*_*fla/che*_*-fliM-GFP spec*	
DK2365	*comI*^*Q12L*^ *Δefp flgM*::*tet*	
DK2777	*comI*^*Q12L*^ *Δefp amyE*::*P*_*hyspank*_*-yeeI spec*	
DK2779	*comI*^*Q12L*^ *Δefp amyE*::*P*_*hyspank*_*-yeeI*^*T19K*^ *spec*	
DK3180	*comI*^*Q12L*^ *Δefp yeeI*^*soe2*^	
DK4092	*comI*^*Q12L*^ *ydiF*::*kan*	
DK4093	*comI*^*Q12L*^ *Δefp ydiF*::*kan*	
DK5168	*comI*^*Q12L*^ *amyE*::*P*_*fla/che*_*-fliY*^*S164A*^*-lacZ cat*	
DK5169	*comI*^*Q12L*^ *Δefp amyE*::*P*_*fla/che*_*-fliY*^*S164A*^*-lacZ cat*	
DK5185	*comI*^*Q12L*^ *amyE*::*P*_*fla/che*_*-fliY-lacZ cat*	
DK5186	*comI*^*Q12L*^ *Δefp amyE*::*P*_*fla/che*_*-fliY-lacZ cat*	
DK5399	*comI*^*Q12L*^ *yacO*::*tet*	
DK5400	*comI*^*Q12L*^ *rae1*::*tet*	
DK5401	*comI*^*Q12L*^ *yacOrae1*::*tet*	
DK5413	*comI*^*Q12L*^ *Δefp yacO*::*tet*	
DK5414	*comI*^*Q12L*^ *Δefp rae1*::*tet*	
DK5415	*comI*^*Q12L*^ *Δefp yacOrae1*::*tet*	
DK5429	*comI*^*Q12L*^ *amyE*::*P*_*hyspank*_*-nusG spec*	
DK5430	*comI*^*Q12L*^ *nusG*::*spec*	
DK5457	*comI*^*Q12L*^ *amyE*::*P*_*hag*_*-lacZ cat*	
DK5458	*comI*^*Q12L*^ *Δefp amyE*::*P*_*hag*_*-lacZ cat*	
DK5512	*comI*^*Q12L*^ *Δefp amyE*::*P*_*hyspank*_*-nusG spec*	
DK5513	*comI*^*Q12L*^ *Δefp nusG*::*spec*	
DK5518	*comI*^*Q12L*^ *Δefp fliY*^*S164A*^	
DK5523	*comI*^*Q12L*^ *Δefp yacO*^*soe11*^	
DK5524	*comI*^*Q12L*^ *Δefp yacO*^*soe15/20*^	
DK5525	*comI*^*Q12L*^ *Δefp yacO*^*soe26*^	
DK5526	*comI*^*Q12L*^ *Δefp rae1*^*soe7*^	
DK5527	*comI*^*Q12L*^ *Δefp rae1*^*soe9*^	
DK5528	*comI*^*Q12L*^ *Δefp rae1*^*soe13*^	
DK5529	*comI*^*Q12L*^ *Δefp ydiF*^*soe22*^	
DK5530	*comI*^*Q12L*^ *Δefp ydiF*^*soe12*^	
DK5531	*comI*^*Q12L*^ *Δefp ydiF*^*soe23*^	
DK5900	*comI*^*Q12L*^ *Δefp ydiF*^*soe16*^	
DK5901	*comI*^*Q12L*^ *Δefp ydiF*^*soe18*^	
DK5955	*comI*^*Q12L*^ *Δefp nusG*^*N21S*^	
DK5995	*comI*^*Q12L*^ *Δefp swrA*::*kan*	
DK6526	*comI*^*Q12L*^ *fliY*^*S164A*^	
DK6533	*comI*^*Q12L*^ *Δefp yeeI*^*soe28*^	
DK6640	*comI*^*Q12L*^ *Δefp fliY*^*S164A*^ *nusG*^*N21S*^	
DK6655	*comI*^*Q12L*^ *efp*::*tet rpsC*^*soe1*^ 26,502 (C > T) 302,890 (A > G)	
DK6656	*comI*^*Q12L*^*efp*::*tet rpsC*^*soe3*^ *nrdI*^*K74E*^	
DK6657	*comI*^*Q12L*^ *Δefp rpsJ*^*soe24*^	
DK6800	*comI*^*Q12L*^ *Δefp rpsJ*^*soe24*^ *nusG*^*N21S*^	
DK7032	*comI*^*Q12L*^ *swrA*::*kan amyE*::*P*_*hag*_*-lacZ cat*	
DK7033	*comI*^*Q12L*^ *Δefp swrA*::*kan amyE*::*P*_*hag*_*-lacZ cat*	
DK7049	*comI*^*Q12L*^ *Δefp fliY*^*S164A*^ *amyE*::*P*_*hag*_*-lacZ cat*	
DK7050	*comI*^*Q12L*^ *Δefp nusG*^*N21S*^ *amyE*::*P*_*hag*_*-lacZ cat*	
DK7073	*comI*^*Q12L*^ *Δefp flgM*::*tet amyE*::*P*_*hyspank*_*-sigD kan*	
DK7074	*comI*^*Q12L*^ *flgM*::*tet amyE*::*P*_*hag*_*-lacZ cat*	
DK7075	*comI*^*Q12L*^ *Δefp flgM*::*tet amyE*::*P*_*hag*_*-lacZ cat*	
DK7076	*comI*^*Q12L*^ *swrA*::*kan flgM*::*tet amyE*::*P*_*hag*_*-lacZ cat*	
DK7077	*comI*^*Q12L*^ *Δefp swrA*::*kan flgM*::*tet amyE*::*P*_*hag*_*-lacZ cat*	
DK7151	*comI*^*Q12L*^ *amyE*::*P*_*yeeI*_*-lacZ cat*	
DK7152	*comI*^*Q12L*^ *amyE*::*P*_*yeeI*_^*soe2*^*-lacZ cat*	
DS322	*flgM*::*tet*	[30]
DS354	*efp*::*tet*	[13]
DS874	*amyE*::*P*_*hyspank*_*-sigD kan*	[33]
DS793	*amyE*::*P*_*hag*_*-lacZ cat*	[33]
DS1639	*swrA*::*kan*	[73]
DS1919	*Δhag amyE*::*P*_*hag*_*-hag*^*T209C*^ *spec*	[34]
DS2197	*pMarA kan mls*	[74]
DS5348	*ΔfliY*	[30]
DS5628	*ΔfliY amyE*::*P*_*hag*_*-hag*^*T209C*^ *spec*	
DS7673	*ΔflgE amyE*::*P*_*fla/che*_*-flgE*^*T123C*^ *cat*	[35]
DS8521	*ΔfliM amyE*::*P*_*fla/che*_*-fliM-GFP spec*	[31]
DS8600	*ΔswrA ΔfliM amyE*::*P*_*fla/che*_*-fliM-GFP spec*	[31]
DS9515	*ΔswrA amyE*::*P*_*hag*_*-hag*^*T209C*^ *spec*	[24]

For quantitative swarm assays, strains were grown to mid log phase (OD_600_ 0.3–1.0) concentrated to an OD_600_ of 10 in PBS pH 7.4 (0.8% NaCl, 0.02% KCl, 100 mM Na_2_HPO_4_, and 17.5 mM KH_2_PO_4_) plus 0.5% India ink. LB plates fortified with 0.65% agar were dried for 10 min open-faced in a laminar flow hood and subsequently inoculated by spotting 10 uL cell resuspensions onto the center of the plate. Plates were dried an additional 10 min open-faced in a laminar flow hood and then incubated at 37°C in a humid chamber. Swarm radius was measured along the same axis every 30 minutes.

Images of swarm plates were obtained by toothpick-inoculating a colony into the center of an LB plate fortified with 0.65% agar. Plates were dried open-faced in a laminar flow hood for 12 min and incubated at 37°C in a humid chamber for 16 hrs. Images were taken using a BioRad Gel Doc.

### Microscopy

Fluorescence micrographs were generated with a Nikon 80i microscope along with a phase contrast objective Nikon Plan Apo 100X and an Excite 120 metal halide lamp. FM4-64 was visualized with a C-FL HYQ Texas Red Filter Cube (excitation filter 532–587 nm, barrier filter >590 nm). GFP and Alexa Fluor 488 were visualized using a C-FL HYQ FITC Filter Cube (FITC, excitation filter 460–500 nm, barrier filter 515–550 nm). Images were captured with a Photometrics Coolsnap HQ2 camera in black and white and subsequently false colored and superimposed using Metamorph image software.

For fluorescent microscopy of flagellar filaments and hooks, 1.0 ml of broth culture was harvested at mid-log phase, resuspended in 50 μl of PBS buffer containing 5μg/ml Alexa Fluor 488 C5 maleimide (Molecular Probes), incubated for 2 min at room temperature, and washed once in 1.0 ml of PBS buffer. The suspension was pelleted, resuspended in 30 μl of PBS buffer containing 5 μg/ml FM 4–64 (Invitrogen T13320), and incubated for 2 min at room temperature. The cells were pelleted, resuspeneded in 30 μl PBS buffer, and were observed by spotting 5 μl of suspension on a microscope slide and immobilized with a poly-L-lysine-treated glass coverslip.

For fluorescent microscopy of flagellar basal bodies, 1.0 ml of broth culture was harvested at mid-log phase, resuspended in 30 μl of PBS buffer containing 5 μg/ml FM 4–64, and incubated for 2 min at room temperature. The cells were pelleted, resuspeneded in 30 μl PBS buffer, and were observed by spotting 5 μl of suspension on a microscope slide and immobilized with a poly-L-lysine-treated glass coverslip.

For super-resolution microscopy, the OMX 3D-SIM Super-Resolution system with a 1.42-numerical-aperture (NA) Olympus 60X oil objective was used. FM4-64 was observed using laser line 561 and emission filter 609 nm to 654 nm, and GFP (along with Alexa Fluor 488) was observed using laser line 488 nm and emission filter 500 nm to 550 nm. Images were captured using PCO Edge 5.5 sCMOS cameras, processed using SoftWorx imaging software, and analyzed using Imaris software.

### Beta-galactosidase assays

Strains were grown in LB at 37°C to OD_600_ 0.7–1.0 and 1 mL was harvested by centrifugation at 18,000 xg. The pellet was resuspended in 1 mL Z-buffer (40 mM NaH_2_PO_4_, 60 mM Na_2_HPO_4_, 10 mM KCl, 1 mM MgSO_4_, and 38 mM 2-mercaptoethanol), 200 μg lysozyme was added, and cells were lysed at 30°C for 15 min. To obtain optical density readings within the linear range, each lysate was appropriately diluted to a final volume of 500 μL in Z-buffer. The reaction was started by the addition of 100 μL start buffer (4 mg/mL ortho-Nitrophenyl-β-galactoside in Z-buffer), and incubated at 30°C. The reaction was stopped by the addition of 250 μL 1M Na_2_CO_3_ and the OD_420_ of the mixture was measured. The β-galactosidase-specific activity was calculated according to the equation (OD_420_ * Dilution factor * 1000) / (time * OD_600_). Average β-galactosidase activity and the standard deviations for all experiments can be found in S10 Table.

### Protein purification and antibody generation

The expression constructs for His-SUMO-FliY (pDP288) and His-SUMO-FliG (pKB43) were introduced into *E. coli* Rosetta gami II cells and grown at 37°C in Terrific broth (12 g tryptone, 24 g yeast extract, 4 ml glycerol, 2.31 g monobasic potassium phosphate and 12.54 g dibasic potassium phosphate per liter) to mid-log phase. 1 mM IPTG was then added and the culture was grown overnight at 16°C. Cells were pelleted, resuspended in lysis buffer (50 mM Na_2_HPO_4_ and 300 mM NaCl) and lysed using an Avestin EmulsiFlex-C3 at approximately 15,000 psi. Cell debris was pelleted by centrifugation at 31,000 xg for 30 min and Ni-nitrolotriacetic acid resin (Novagen) was added to the clarified supernatant. The resin-lysate mixture was incubated at 4°C for 3 hrs. The resin was applied to a 1-cm separation column (Bio-Rad), washed twice with 10 mL lysis buffer, and once with 10 mL wash buffer (50 mM Na2HPO4, 300 mM NaCl, and 30 mM imidazole) Protein was eluted with lysis buffer containing 100 mM imidazole. To cleave the His-SUMO tag from the purified protein, ubiquitin ligase/protease was added and the reaction was incubated at room temperature for 3 hrs. To remove remaining uncleaved protein or free His-SUMO from the cleavage reaction, Ni-nitrolotriacetic acid resin (Novagen) was added and incubated at 4°C for 1 h. The resin was pelleted by centrifugation and the supernatant, containing untagged FliY or FliG, was dialyzed into PBS pH 7.4 plus 10% glycerol and stored at -20°C.

One milligram of purified FliY protein was sent to Cocalico Biologicals Inc. for serial injection into a rabbit host for antibody generation. Anti-FliY serum was mixed with FliY-conjugated Affi-Gel-10 resin (Bio-Rad 1536099) and incubated overnight at 4°C. The resin was packed onto a 1-cm column (Bio-Rad) and then washed with 100 mM glycine (pH 2.5) to release the antibody and immediately neutralized with 2M Tris base. The purification of the antibody was verified by SDS-PAGE. Purified anti-FliY antibody was dialyzed into PBS–50% glycerol and stored at -20°C.

### Ribosome profiling library construction

Ribosome profiling libraries were prepared as described previously with minor modifications [75]. 300 mL LB exponential phase cultures (OD_600_ 0.3–0.4) grown at 37°C were subjected to rapid filtration and subsequently flash frozen in liquid nitrogen. Cells were lysed in 650 μL lysis buffer (10 mM MgCl_2_, 100 mM NH_4_Cl, 5 mM CaCl_2_, 20 mM Tris pH 8.0, 0.1% NP-40, 0.4% Triton X-100, 0.1 units/μL RNase free DNase I (Invitrogen AM2222), 0.5 units/μL Superase-In (Invitrogen AM2696)) using a Spex 6875 freezer mill set to 10 cycles of 2 min runs at 15 cps separated by 2 min rests. Following lysis, 25 A260 units of lysate were digested with 1500 units of S7 micrococcal nuclease (Roche 10107921001) for 1 hr at room temp after which the reaction was quenched by the addition of EGTA to a final concentration of 6 mM. The digested lysate was then applied to a 10%-50% sucrose gradient and centrifuged in a Ti-40 rotor at 35,000 rpm for 2.5 hrs at 4°C. 700 μL of fractions containing 70S ribosomes were denatured in 1% SDS and extracted once with an equal volume of 75°C acid phenol, once with an equal volume of room temp acid phenol, and RNA was precipitated with isopropanol. The precipitant was resuspended in 12 μL H2O and 25 μg RNA was mixed with 2X loading dye (10 mM EDTA, 30 μg/mL bromophenol blue, and 98% formamide) and resolved on a 15% polyacrylamide TBE Urea gel. After staining the gel in SYBR Gold (Sigma S11494) for 3 min, products between ~15 and 40 bp were excised using the 10 bp O’range ladder as the standard (Thermo Scientific SM1313) and subsequently gel extracted. RNA was resuspended in H_2_O and the 3’ ends were dephosphorylated with T4 poly-nucleotide kinase (Lucigen 30061–1) at 37°C for 1 hr. RNA was precipitated in isopropanol and ligated to 1 μL 1 μg/μL Linker 1 (IDT /5rApp/CTGTAGGCACCATCAAT/3ddC/) with T4 RNA Ligase 2, truncated (NEB M0242S) in a 50 μL reaction at 25°C for 2.5 hrs. Products were precipitated, mixed in 2X loading dye and resolved on a 15% polyacrylamide TBE Urea gel. After staining the gel in SYBR Gold for 3 min, products between 30 and 100 bp were excised using the 10 bp O’range ladder as the standard and subsequently gel extracted. Isolated RNA was then reverse transcribed using Superscript III (Invitrogen 18080044) and 2 μL 1.25 μM reverse transcription primer (IDT 5′-(Phos)-AGATCGGAAGAGCGTCGTGTAGGGAAAGAGTGTAGATCTCGGTGGTCGC-(SpC18)-CACTCA-(SpC18)-TTCAGACGTGTGCTCTTCCGATCTATTGATGGTGC CTACAG-3′) in a 20 μL reaction for 30 min at 48C. RNA was subsequently hydrolyzed by the addition of 2.2 μL 1N NaOH and incubation at 98C for 20 min. Reverse transcription products were resolved on a 10% polyacrylamide TBE urea gel, the gel was stained in SYBR Gold for 3 min, and cDNA products were gel extracted. After resuspension in H_2_O, cDNA products were circularized using CircLigase (Epicentre CL4111K) in a 20 μL reaction volume at 60°C for 1 hr and subsequently heat-inactivated at 80°C for 10 min. Circularized products were precipitated in isopropanol, resuspended in H_2_O, and used as a template for 20 μL PCR reactions using Phusion Polymerase (NEB M0530S) with forward library primer (IDT 5′-AATGATACGGCGACCACCGAGATCTACAC-3′) and Indexed reverse library primer (IDT 5′-CAAGCAGAAGACGGCATACGAGATNNNNNNNNGACTGGAGTTCAGACGTGTGCTCTTCCG-3′) where NNNNNNNN represents the barcode sequence unique to each library. After 6–10 cycles, two PCR reactions per sample with no apparent duplexed products after resolution of 2 μL on an 8% polyacrylamide TBE urea gel were pooled and DNA was purified with a QIAquick kit (Qiagen 28106) and eluted in 20 μL H_2_O. Libraries were sequenced using the Illumina NextSeq 500 platform in a single-end flow cell at the Indiana University Center for Genomics and Bioinformatics.

### RNA sequencing library construction

Total RNA was extracted from the same cell lysates used to create ribosome profiling libraries. Following lysis, 2.5 A260 units were diluted in 700 μL H_2_O and denatured in 1% SDS. RNA was extracted once with an equal volume of hot acid phenol, once with an equal volume of room temp acid phenol, and RNA was precipitated with isopropanol. Precipitant was resuspended in H_2_O and 10 μg RNA was DNAse treated using 4 units of RNase free DNase I (Invitrogen AM2222) at 37°C for 30 min in a 20 μL reaction volume. RNA was precipitated in isopropanol, resuspended in H_2_O and libraries were prepared by the Indiana University Center for Genomics and Bioinformatics using the ScriptSeq RNA-library kit (Illumina SSV21124). Libraries were sequenced using the Illumina NextSeq 500 platform in a single-end flow cell at the Indiana University Center for Genomics and Bioinformatics.

### Ribosome profiling data analysis

NGSutils v 0.5.9 was used to remove sequencing adapters (CTGTAGGCACCATCAAT) and filter out any reads shorter than 25 bp. Fastx v 0.0.13 was subsequently used to remove the first base from each read and resulting reads were aligned to the NCIB 3610 genome (NZ_CP020102.1) using Bowtie v 1.1.2. Using the 3primeassignment.pl script (S1 File), 1,750,000–3,550,000 reads per sample that uniquely aligned to the genome were assigned to a single position corresponding to the 15^th^ nucleotide from the 3’ end according to the 3’ assignment method described previously [10]. Only genes with an average read density greater than 0.1 (defined as the number of mapped reads divided by the number of codons) in all samples were analyzed further (S11 Table). For each sample, the pausescore.pl script (S1 File) was used to determine the pause score for each codon in the filtered list of genes defined as the number of reads assigned to that position divided by the average read density of that gene. The first and last 6 codons of each gene were excluded from this analysis. The pause scores for all codons calculated in this way can be found in S3 Table. Average pause scores for all 8,000 potential tripeptides were determined in one of two ways–either with the tripeptide centered on the P-site or the E-site. In both methods only the pause score for the P-site codon was used to determine the average. The average pause scores for all tripeptides calculated in this way can be found in S5 Table. Weighted sequence logos were generated by compiling all sequences in which the P-site codon had a pause score of 10 or greater and visualized using the WebLogo 3 online tool at http://weblogo.threeplusone.com/. Clustered orthologous group assignment was performed with the DIAMOND mapping mode of eggNOG version 4.5 [76].

*E*. *coli* ribosome profiling datasets (SRX823699, SRX823700, SRX823701, and SRX823703) published by Woolstenhulme et al., 2015 were downloaded from the Sequence Read Archive and analyzed as described above using the MG1655 genome (NC_000913.3) as a reference.

### RNA sequencing data analysis

RNA-sequencing analysis was performed using the default parameters of the RSEM (v 1.3.0) calculate expression function and the NCIB 3610 genome (NZ_CP020102.1) as a reference [77]. The transcripts per kilobase million (TPM) reported in the RSEM output were used to generate Fig 8 and S8 Table.

### FliY Sequence Logo Generation

A local database of 2554 genomes were annotated with the Pfam library using the software hmmer v 3.1b2 and an E-value threshold of 1e-10 [78,79]. Proteins that contained both a CheC and FliMN_C domain were considered to be FliY homologs. The resulting 282 sequences were aligned using the default parameters of muscle version 3.8.31 [80] and visualized using the WebLogo 3 online tool at http://weblogo.threeplusone.com/. The list of FliY homolog accession numbers can be found in S1 Table.

### Quantitative Western blot analysis

Strains were grown to mid-log phase, concentrated to an OD_600_ of 10 in lysis buffer (17.2 mM Tris pH 7.0, 8.6 mM EDTA pH 8.0, 1 mg/ml Lysozyme, 0.1 mg/ml RNaseA, 20 mg/ml DNase I and 50 mg/ml phenylmethane sulfonyl fluoride) and incubated at 37°C for 30 min. 6X SDS sample buffer (500 mM Tris pH 6.8, 22% glycerol, 10% SDS and 0.12% bromophenol blue) was added, and samples were boiled for 5 min. 12 μL boiled samples containing 2.13x10^7^ cells (calculated according to the equation: 2.13x10^8^ cells/mL/OD_600_) were loaded onto 12% polyacrylamide gels. To generate the standard curves, the following amounts of protein were mixed with 12 uL of the corresponding deletion mutant’s lysate and the entire mixtures were loaded onto each appropriate gel: FliY– 0.184 pmol, 0.092 pmol, 0.037 pmol, 0.018 pmol, or 0.0092 pmol. FliG– 0.22 pmol, 0.144 pmol, 0.072 pmol, 0.029 pmol, or 0.014 pmol. Lysates were resolved at 150 V for 1.25 h and transferred onto nitrocellulose membranes. For blots in which FliY concentration was analyzed, a 1:2,000 dilution of affinity purified anti-FliY antisera and a 1:2,000 dilution of affinity purified anti-FliG antisera (serving as a loading control) were used as primary antibodies. For blots in which FliG concentration was analyzed, a 1:2,000 dilution of affinity purified anti-FliG antisera and a 1:80,000 dilution of crude anti-σ^A^ antisera (serving as a loading control) were used as primary antibodies. Following incubation with the primary antibodies, nitrocellulose membranes were probed with Alexa Fluor 750-conjugated goat anti-rabbit immunoglobulin G (Life technologies A21039) and blots were imaged using a FluorChem R system. Images were analyzed using Image Studio Lite version 5.2.

### Phylogenetic distribution of Soe proteins

To determine the conservation of S3, S10, NusG, YeeI, YacO, and Rae1 across all domains of life, the genomes of 191 organisms identified by Ciccarelli et al. were annotated with the Pfam library using the software hmmer v 3.1b2 and an E-value threshold of 1e-5 [78,79,81]. The presence of S10 was established by the annotation of a Ribosomal_S10 domain. The presence of S3 was established by the annotation of a protein that contained both a Ribosomal_S3_C domain as well as a KH_2 domain. The presence of NusG was established by the annotation of a protein that contained both a KOW domain as well as a NusG domain. The presence of YeeI was established by the annotation of a Transcrip_reg domain. The presence of YacO was established by the annotation of a protein that contained both a SpoU_methylase domain as well as a SpoU_sub_bind domain. Finally, the presence of Rae1 was established by the annotation of a NYN_YacP domain. To determine the conservation of YdiF, a blastp database consisting of all YdiF-subfamily sequences identified previously was constructed [43]. The genomes of all 191 organisms were then analyzed for sequences that aligned to at least one YdiF homolog with an E-value threshold of 1e-10 and greater than 75% identity using BLAST+ version 2.2.31 [82]. The data are presented using the Interactive Tree of Life visualization software [83]. All accession numbers for the identified homologs can be found in S7 Table.

### Data and software availability

Ribosome profiling and RNA-sequencing data are available at the Gene Expression Omnibus under accession number GSE126235. Data were analyzed using custom Perl scripts.

## Supporting information

S1 Text Supplementary methods and references. Methods describing strain construction and suppressor isolation as well as supplementary references.
(PDF)

S1 File Custom Perl scripts used to analyze the ribosome profiling dataset. (TAR)

S1 Fig Quantitative swarm assays. Quantitative swarm expansion assays in which mid-log phase cultures were concentrated and used to inoculate swarm agar plates. Swarm expansion was monitored along the same axis every 30 min for 6.5 hrs. Each data point represents the average of three replicates and the raw values can be found in S2 Table. The following strains were used as the inoculum: A) *soe2* (DK3180) and *soe28* (DK6533). B) *soe11* (DK5523), *soe15* (DK5524), *soe26* (DK5525), *soe9* (DK5527), *soe13* (DK5528), and *soe7* (DK5526). C) *soe16* (DK5900), *soe22* (DK5529), *soe23* (DK5531), *soe12* (DK5530), and *soe18* (DK5901). D) *efp nusG* (DK5513). E) *efp flgM* (DK2365), and *efp flgM P*_*IPTG*_*-sigD* (DK7073). F) WT (DK1042) and *fliY*^*S164A*^ (DK6526).
(PDF)

S2 Fig Soe proteins are broadly distributed across the tree of life. The distribution of homologs of the Soe proteins S10 (dark blue), S3 (black), NusG (red), YeeI (gold), YacO (green), Rae1 (purple), and YdiF (cyan) across the three domains of life. Numbers indicate the following clades (1) Flavobacterium-Cytophaga-Bacteroides group, (2) Chlamydiales, (3) Planctomycetes, (4) Spirochaetes, (5) Actinobacteria, (6) Deinococcus-Thermus group and (7) Cyanobacteria. *Bacillus subtilis* is highlighted in pink. S7 Table contains the homolog accession numbers for each species. We note that an S3 homolog was not detected in 3 genomes analyzed. Due to the presence of S3 homologs in close relatives of those strains, we predict that this is due to incomplete genome annotation.
(PDF)

S3 Fig *soe2* results in an increase in *P_yeeI_* activity. β-galactosidase activity reported in Miller Units (MU) of transcriptional fusions of *lacZ* to *P*_*yeeI*_ *and P*_*yeeI*_^*soe2*^. Error bars indicate the standard deviation of 3 biological replicates and the raw values can be found in S2 Table. The following strains were used to generate this panel: *P*_*yeeI*_*-lacZ* (DK7151), *P*_*yeeI*_^*soe2*^ (DK7152).
(PDF)

S4 Fig *Escherichia coli efp* mutants have increased ribosome pausing in ValS and FliN. The data used to generate this figure are derived from GEO accession number GSE64488. Panels A, B) Average ribosome profiling pause scores of each codon within the ValS open reading frame. The position of the PPP motif is indicated by a red asterisk on the X-axis. Panels C, D) Weighted sequence logos of amino acid sequences in which the P-site codon had a pause score of 10 or greater in the ribosome profiling datasets from WT or *efp*. Panels E, F) Average ribosome profiling pause scores of each codon within the FliN open reading frame. The box indicates the location of the VVVV motif.
(PDF)

S5 Fig EF-P alleviates ribosome pausing in the *B. subtilis* valine tRNA synthetase, ValS. Panels A, C) Average ribosome profiling pause scores of each codon within the ValS open reading frame. The position of the PPP motif is indicated by a red asterisk on the X-axis. Panels B, D) Average pause scores for ValS codons 28–58. The box indicates the location of the PPP motif. Error bars indicate standard deviation of 3 biological replicates. The following strains were used to generate this figure: WT (DK1042), and *efp* (DK2050).
(PDF)

S1 Table FliY homologs. List of the accession numbers of FliY homologs used to generate Fig 1C.
(XLSX)

S2 Table Numerical values associated with graphs. (Sheet 1) Average swarm radius in mm over time for all reported swarm assays. (Sheet 2) Hook and basal body quantifications and associated cell lengths reported in Fig 2. (Sheet 3) Average number of FliY and FliG molecules per cell reported in Fig 6. (Sheet 4) Average β-galactosidase activity reported in Miller Units (MU) and the standard deviations for all reported β-galactosidase assays.
(XLSX)

S3 Table *B. subtilis* ribosome pause sites. Pause scores of codons in all genes with greater than 0.1 read per codon. The “position” field corresponds to the nucleotide position of the first base in the codon within the NCIB 3610 genome.
(XLSX)

S4 Table EF-P-alleviated ribosome pause sites. Ribosome pause sites in which a 10-fold or greater pause score was observed in the *efp* mutant compared to wild type. Whether or not each gene is reported as essential is indicated. The “position” field corresponds to the nucleotide position of the first base in the codon within the NCIB 3610 genome.
(XLSX)

S5 Table *B. subtilis* tripeptide pause scores. Average pause scores of all 8,000 tripeptides centered on the P-site (sheet 1) or E-site (sheet 2) and the number of each tripeptide’s occurrences in genes with greater than 0.1 read per codon. In all cases, only the pause score of the P-site codon was used to calculate the average.
(XLSX)

S6 Table EF-P-alleviates ribosome pausing in proteins of diverse function. Quantification of the number of occurrences (#) and corresponding percent (%) of each cluster of orthologous groups within genes containing EF-P alleviated ribosome pause sites and all genes encoded on the NCIB 3610 chromosome.
(DOCX)

S7 Table Soe protein conservation. List of the accession numbers identified to be homologous to S10, S3, NusG, YeeI, YacO, Rae1, and YdiF in 191 genomes across the tree of life. These data were used to generate S2 Fig.
(XLSX)

S8 Table RNA-sequencing summary. Transcripts per kilobase million (TMP) quantifications of each gene in the NCIB 3610 genome.
(XLSX)

S9 Table Plasmids used in this study. (DOCX)

S10 Table Primers used in this study. (DOCX)

S11 Table Average ribosome densities within genes analyzed in this study. Average ribosome densities were determined by the number of reads mapping to a particular gene divided by the number of codons within that gene. Only genes in with an average ribosome density greater than 0.1 in all 12 ribosome profiling libraries were analyzed.
(XLSX)
