# Supplementary material for: Suppressor mutations in ribosomal proteins and FliY restore Bacillus subtilis swarming motility in the absence of EF-P
Source: PLoS Genet. 2019 Jun 25;15(6):e1008179. doi: 10.1371/journal.pgen.1008179 (PMC6613710; doi:10.1371/journal.pgen.1008179)
Supplement: S1 Text — Methods describing strain construction and suppressor isolation as well as supplementary references. (PDF) [file pgen.1008179.s001.pdf]

SUPPLEMENTARY METHODS

Strain Construction. *SPP1-mediated transduction.* SPP1-mediated transductions were performed as described previously (Yasbin, 2004). In short, lysates were created on *B. subtilis* strains grown in TY (1% Tryptone, 0.5% yeast extract, 0.5% NaCl, 10mM MgSO4, and 1mM MnSO4). Recipient strains were grown in TY to stationary phase, 1 mL diluted into 9 mL TY, and 10 µL (for tetracycline selection) or 25 µL (for spectinomycin and kanamycin selection) lysates were added, followed by incubation at room temperature for 30 min and selection for the respective antibiotic resistance at 37°C overnight. For transductions in which spectinomycin or kanamycin-resistance was selected for, 10 mM sodium citrate was added to the selection plates.

$P_{hyspank}$-*yeeI*. The *yeeI* open reading frame was amplified from 3610 or soe29 chromosomal DNA using primer pair 4409/4410 and subsequently ligated into the NheI/SphI restriction sites of pDR111 to create pKRH24 and pKRH25, respectively. pKRH24 and pKRH25 were then transformed into DK2050 to create DK2777 and DK2779, respectively.

$P_{hyspank}$-*nusG*. The nusG open reading frame was amplified from 3610 chromosomal DNA using primer pair 5978/5979 and subsequently ligated into the NheI/SphI restriction sites in pDR111 to create pKRH116. pKRH116 was then transformed into DK1042 to produce DK5429. The *amyE::*$P_{hyspank}$*-nusG spec* construct was subsequently introduced into DK2050 to produce DK5512.

Δ*fliY.* Deletion of *fliY* was achieved through allelic replacement. Plasmid pSG6 (Calvo, 2015) was passaged through the recA+ *Escherichia coli* strain TG1, transformed into DK1042, integrated by selecting mls resistant colonies at 37°C, and evicted by

passage at room temperature. Mls-sensitive colonies were isolated and confirmed to encode the deletion by PCR length polymorphism analysis.

Δ*fliMY*. Deletion of *fliMY* was achieved through allelic replacement. 3610 genomic DNA was amplified using primer pairs 4135/4136 and 4137/4138 and the resulting fragments were introduced into the SmaI restriction site of pMiniMAD2 using Gibson assembly (Gibson, 2009) to produce pKRH9. Plasmid pKRH9 was passaged through the recA+ *Escherichia coli* strain TG1, transformed into DK1042, integrated by selecting mls resistant colonies at 37°C, and evicted by passage at room temperature. Mls-sensitive colonies were isolated and confirmed to encode the deletion by PCR length polymorphism analysis.

ydiF::kan. Mutation of *ydiF* was performed by amplifying the regions upstream (with primers 5073/5074) and downstream (with primers 5075/5076) of *ydiF* using 3610 chromosomal DNA as a template, and amplifying the kanamycin resistance cassette from pDG780 with primers 3250/3251 (Geurot-Fleury, 1995). The three fragments were ligated by Gibson assembly and transformed into DK1042 to produce DK4092. The mutation was confirmed by PCR length polymorphism analysis. The *ydiF::kan* mutation from DK4092 was subsequently introduced into DK2050 by SPP1-mediated transduction to produce DK4093.

yacO::tet. Mutation of *yacO* was performed by amplifying the regions upstream (with primers 5077/5078) and downstream (with primers 5079/5080) of *yacO* using 3610 chromosomal DNA as a template, and amplifying the tetracycline resistance cassette from pDG1515 with primers 3250/3251 (Geurot-Fleury, 1995). The three fragments were ligated by Gibson assembly and transformed into DK1042 to produce DK5399.

The mutation was confirmed by PCR length polymorphism analysis. The *yacO::tet* mutation from DK5399 was subsequently introduced into DK2050 by SPP1-mediated transduction to produce DK5413.

rae1::tet. Mutation of *rae1* was performed by amplifying the regions upstream (with primers 5081/5082) and downstream (with primers 5083/5084) of *rae1* using 3610 chromosomal DNA as a template, and amplifying the tetracycline resistance cassette from pDG1515 with primers 3250/3251 (Geurot-Fleury, 1995). The three fragments were ligated by Gibson assembly and transformed into DK1042 to produce DK5400. The mutation was confirmed by PCR length polymorphism analysis. The *rae1::tet* mutation from DK5400 was subsequently introduced into DK2050 by SPP1-mediated transduction to produce DK5414.

yacOrae1::tet. Simultaneous mutation of *yacO* and *rae1* was performed by amplifying the regions upstream of *yacO* (with primers 5077/5078) and downstream of *rae1* (with primers 5083/5084) using 3610 chromosomal DNA as a template, and amplifying the tetracycline resistance cassette from pDG1515 with primers 3250/3251 (Geurot-Fleury, 1995). The three fragments were ligated by Gibson assembly and transformed into DK1042 to produce DK5401. The mutation was confirmed by PCR length polymorphism analysis. The *yacOrae1::tet* mutation from DK5401 was subsequently introduced into DK2050 by SPP1-mediated transduction to produce DK5415.

nusG::spec. Mutation of *nusG* was performed by amplifying the regions upstream (with primers 5968/5969) and downstream (with primers 5970/5971) of *nusG* using 3610 chromosomal DNA as a template, and amplifying the spectinomycin resistance

cassette from pAH54 with primers 3250/3251 (Geurot-Fleury, 1995). The three fragments were ligated by Gibson assembly and transformed into DK1042 to produce DK5430. The mutation was confirmed by PCR length polymorphism analysis. The *nusG::spec* mutation from DK5430 was subsequently introduced into DK2050 by SPP1-mediated transduction to produce DK5513.

His-SUMO fusions. The *fliY* and *fliG* open reading frames were amplified from *B. subtilis* 3610 chromosomal DNA with primer pairs 1295/1296 and 882/883, respectively. The resulting fragments were introduced into the SapI/XhoI restriction sites in pTB146 to produce pDP288 (His-SUMO-FliY) and pKB43 (His-SUMO-FliG).

FliY-lacZ translational reporter. The *fliY-lacZ* fusion constructs were made by amplifying the *fliY* locus with primer pair 869/5894 from either 3610 or *soe8* chromosomal DNA and amplifying the $P_{fla/\mathrm{che}}$ promoter with primer pair 2015/322 from 3610 chromosomal DNA. The resulting $P_{fla/\mathrm{che}}$ fragment as well as *fliY* or $fliY^{S164A}$ fragments were ligated into the EcoRI/SalI restriction sites of pDG1728 to produce pKRH91 and pKRH94, respectively. pKRH91 was transformed into DK1042 and DK2050 to produce DK5185 and DK5186, respectively. pKRH94 was transformed into DK1042 and DK2050 to produce DK5168 and DK5169, respectively. Integration of the plasmids at the *amyE* locus was verified by the inability of the isolates to degrade starch.

P_{yeeI}-lacZ transcriptional reporter. The P_{yeeI}-*lacZ* fusion constructs were made by amplifying the *yeeI* promoter with primer pair 4406/4408 from either 3610 or *soe2* chromosomal DNA and ligating the resulting fragments into the EcoRI and HindIII restriction sites of pDG268 to produce pKRH180 and pKRH181, respectively. pKRH180

and pKRH181 were transformed into DK1042 to produce DK7151 and DK7152, respectively and the integration of the plasmids at the amyE locus was verified y the inability of the isolates to degrade starch.

Suppressor isolation and identification. *Suppressor isolation*. LB plates fortified with 0.7% agar were dried open-faced in a laminar flow hood for 10 min, centrally inoculated with either DK2050 or DS354, and subsequently dried an additional 10 min open-faced in a laminar flow hood. Plates were incubated at 37C in a humid chamber until a flare of swarming competent cells emerged from the site of inoculation (20-48 hrs). Approximately 95% of plates produced at least one suppressor flare within this time period. For each plate in which swarming suppressors were apparent, a single colony was isolated from the edge of the swarm front, verified to contain the *efp* deletion construct, and the ability to swarm was confirmed by quantitative swarm assay.

Transposon-linked SPP1 generalized phage transduction mapping. pMarA was introduced into *soe2* via by SPP1-mediated phage transduction followed by selection for mls resistance at room temperature. pMarA contains a transposon encoding a kanamycin resistance cassette (TnYLB) and a temperature-sensitive origin that allows for replication at room temperature but not 42°C in B. subtilis. Three of the resulting colonies were separately inoculated into 3 mL LB + mls cultures and transposon mutagenesis was allowed to occur by incubation at room temperature overnight. Mutants with transposon insertions in the genome were selected by incubating cells at 42°C on LB plates containing kanamycin. Approximately 1,000-4,000 colonies from each pool were combined and used to create a SPP1 lysate as described above. The

resulting lysates were transduced into DS354 and kanamycin resistance was selected. Approximately 1,000 colonies per pool were combined and each pool was used to inoculate separate swarming motility agar plates. Following a 5- to 9-h incubation at 37°C, swarming proficient mutants emerged from the site of inoculation as a disk of motile cells, and one colony per pool was isolated from the edge of the swarm flare. To confirm that the transposon was linked to the suppressor mutation, a lysate was generated on the suppressor mutant and the transposon was transduced to the parent strain lacking the suppressor. Three hundred of the resulting colonies were then picked onto 0.7% LB swarm agar plates to enumerate the number of colonies with motile cells. The percentage of colonies that were motile was inversely proportional to the distance between the transposon and suppressor mutation.

To determine the locations of the transposon insertion sites, genomic DNA was isolated from each isolate, digested with Sau3A1, and ligated using T4 ligase to create circular fragments. Primer pair 695/696, which anneals to TnYLB and directs polymerization outwards from the transposon was used to PCR amplify the neighboring DNA. The resulting DNA fragments were subsequently sequenced with primer 696 to determine the transposon insertion site.

Whole genome sequencing. *Soe1, 3, 8, 11, 12, 13, 16, 17, 24* and DK2050 chromosomal DNA was used to prepare Illumina sequencing libraries using a Nextera DNA XT kit. Paired-end sequencing was performed on a MiSeq platform with 500 cycles at TUCF genomics. The resulting reads were aligned to the NCIB 3610 genome (NZ_CP020102.1) and mutations were called using Breseq v 2.1.0 (REF).

Sequencing yeeI, ydiF, yacO, and rae1 loci. A PCR product containing the *yeeI* open reading frame was amplified from *B. subtilis* chromosomal DNA (either from DS354, DK2050, or the appropriate suppressor strain) using primer pair 4346/4347. The *yeeI* PCR was then sequenced using primers 4349, 4350, or 4351 individually.

A PCR product containing the *ydiF* open reading frame was amplified from *B. subtilis* chromosomal DNA (either from DS354, DK2050, or the appropriate suppressor strain) using primer pair 5085/5072. The *ydiF* PCR was then sequenced using primers 5085, 5086, 5087, or 5072 individually.

A PCR product containing the *yacO* and *rae1* open reading frames was amplified from *B. subtilis* chromosomal DNA (either from DS354, DK2050, or the appropriate suppressor strain) using primer pair 5088/5090. The resulting PCR was then sequenced using primers 5088, 5089, or 5090 individually.

yacO soe reconstruction. Site-directed mutation of *yacO* was achieved through allelic replacement. The *yacO* open reading frame was amplified with primer pair 6019/6020 using *soe11*, *soe15*, or *soe26* chromosomal DNA as a template and subsequently ligated into the EcoRI/BamHI restriction sites of pMiniMad2 to create pKRH133, pKRH134, and pKRH135, respectively. The resulting plasmids were passaged through the recA+ *Escherichia coli* strain TG1, transformed into DK2050, integrated by selecting mls resistant colonies at 37°C, and evicted by passage at room temperature. Mls-sensitive colonies were isolated and the *yacO* locus was sequenced to determine the allele present at that site.

rae1 soe reconstruction. Site-directed mutation of *rae1* was achieved through allelic replacement. The *rae1* open reading frame was amplified with primer pair

6021/6022 using *soe7*, *soe9*, or *soe13* chromosomal DNA as a template and subsequently ligated into the EcoRI/BamHI restriction sites of pMiniMad2 to create pKRH136, pKRH137, and pKRH138, respectively. The resulting plasmids were passaged through the recA+ *Escherichia coli* strain TG1, transformed into DK2050, integrated by selecting mls resistant colonies at 37°C, and evicted by passage at room temperature. Mls-sensitive colonies were isolated and the *rae1* locus was sequenced to determine the allele present at that site.

ydiF soe reconstruction. Site-directed mutation of *ydiF* was achieved through allelic replacement. The *ydiF* open reading frame was amplified with primer pair 6023/6024 using *soe12*, *soe16*, *soe18*, *soe22*, or *soe23* chromosomal DNA as a template and subsequently ligated into the BamHI/SalI restriction sites of pMiniMad2 to create pKRH139, pKRH140, pKRH141, pKRH142, and pKRH143, respectively. The resulting plasmids were passaged through the recA+ *Escherichia coli* strain TG1, transformed into DK2050, integrated by selecting mls resistant colonies at 37°C, and evicted by passage at room temperature. Mls-sensitive colonies were isolated and the *ydiF* locus was sequenced to determine the allele present at that site.

yeeI soe reconstruction. Site-directed mutation of *yeeI* was achieved through allelic replacement. The *yeeI* locus was amplified form *soe2* chromosomal DNA with primer pair 4411/4412 or from *soe29* chromosomal DNA with primer pair 4401/4412 and the resulting fragments were introduced into the SmaI restriction site of pMiniMAD2 using Gibson assembly (Gibson, 2009) to produce pKRH20 and pKRH28, respectively. The resulting plasmids were passaged through the recA+ *Escherichia coli* strain TG1, transformed into DK2050, integrated by selecting mls resistant colonies at 37°C, and

evicted by passage at room temperature. Mls-sensitive colonies were isolated and the *yeeI* locus was sequenced to determine the allele present at that site.

nusG^{N21S} reconstruction. Site-directed mutation of *nusG* was achieved through allelic replacement. The *nusG* open reading frame was amplified with primer pair 5968/6025 using *soe17* chromosomal DNA as a template and subsequently ligated into the EcoRI/BamHI restriction sites of pMiniMad2 to create pKRH144. pKRH144 was passaged through the recA+ *Escherichia coli* strain TG1, transformed into DK2050, DK5518, or DK6657 integrated by selecting mls resistant colonies at 37°C, and evicted by passage at room temperature. Mls-sensitive colonies were isolated and the *nusG* locus was sequenced to determine the allele present at that site.

fliY^{S164A} reconstruction. Site-directed mutation of *fliY* was achieved through allelic replacement. The *fliY* open reading frame was amplified using primer pair 1703/1704 or primer pair 1703/6446 using *soe8* chromosomal DNA as a template and subsequently ligated into the BamHI/SalI restriction sites of pMiniMad2 to create pKRH145 and pKRH165, respectively. The resulting plasmids were passaged through the *recA+ E. coli* strain TG1. pKRH145 was transformed into DK2050 and pKRH165 was transformed into DK1042, integrated by selecting mls resistant colonies at 37°C, and evicted by passage at room temperature. Mls-sensitive colonies were isolated and the *fliY* locus was sequenced to determine the allele present at that site.

SUPPLEMENTARY REFERENCES

Antoniewski, C., Savelli, B., and Stragier, P. (1990) The *spoIIJ* gene, which regulates early developmental steps in *Bacillus subtilis*, belongs to a class of environmentally responsive genes. *J Bacteriol*. **172**(1):86-93.

Bendezú, F. O., Hale, C. A., Bernhardt, T. G., and de Boer, P. A. (2009) RodZ (YfgA) is required for proper assembly of the MreB actin cytoskeleton and cell shape in *E. coli*. *EMBO J.* **28**:193-204.

Guérout-Fleury, A.M., Shazand, K., Frandsen N., and Stragier, P. (1995) Antibiotic-resistance cassettes for *Bacillus subtilis*. *Gene* **167**:335-336.

Guérout-Fleury, A.M., Frandsen, N., and Stragier, P. (1996) Plasmids for ectopic integration in *Bacillus subtilis.* *Gene* **180**:57-61.

Patrick, J.E., and Kearns, D.B. (2008) MinJ (YvjD) is a topological determinant of cell division in *Bacillus subtilis*. *Mol Microbiol* **70**:1166-1179.
