# Supplementary material for: Suppressor mutations in ribosomal proteins and FliY restore Bacillus subtilis swarming motility in the absence of EF-P
Source: PLoS Genet. 2019 Jun 25;15(6):e1008179. doi: 10.1371/journal.pgen.1008179 (PMC6613710; doi:10.1371/journal.pgen.1008179)
Supplement: S1 Fig — Quantitative swarm expansion assays in which mid-log phase cultures were concentrated and used to inoculate swarm agar plates. Swarm expansion was monitored along the same axis every 30 min for 6.5 hrs. Each data point represents the average of three replicates and the raw values can be found in S2 Table. The following strains were used as the inoculum: A) soe2 (DK3180) and soe28 (DK6533). B) soe11 (DK5523), soe15 (DK5524), soe26 (DK5525), soe9 (DK5527), soe13 (DK5528), and soe7 (DK5526). C) soe16 (DK5900), soe22 (DK5529), soe23 (DK5531), soe12 (DK5530), and soe18 (DK5901). D) efp nusG (DK5513). E) efp flgM (DK2365), and efp flgM PIPTG-sigD (DK7073). F) WT (DK1042) and fliYS164A (DK6526). (PDF) [file pgen.1008179.s003.pdf]

Supplementary Figure 1

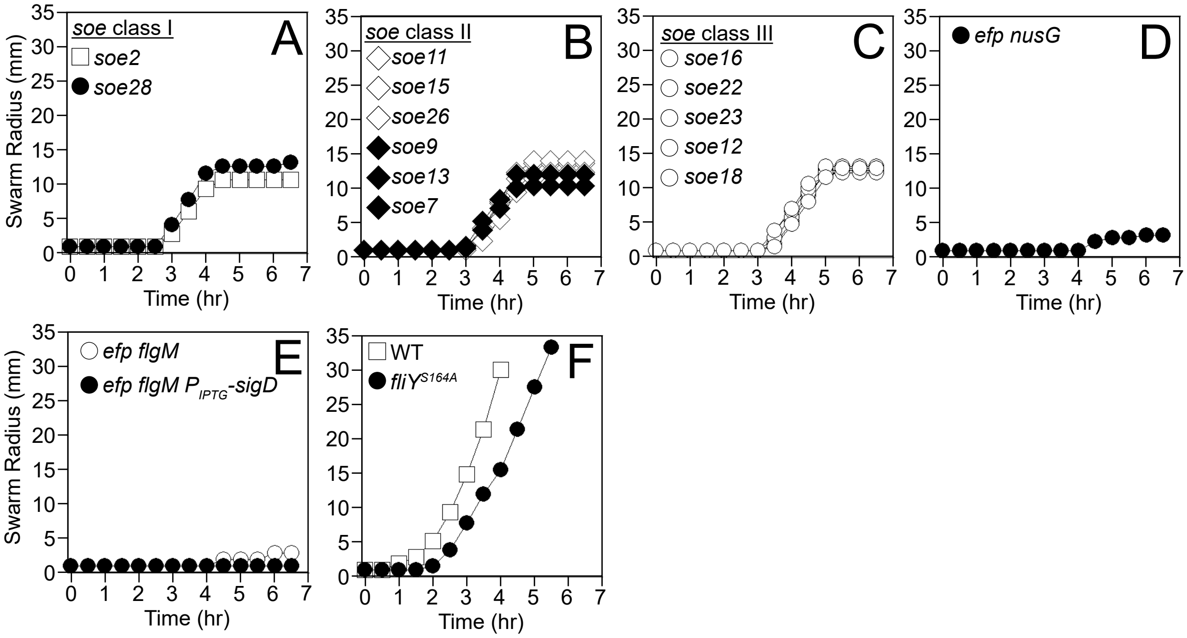

Supplementary Figure 1. Quantitative Swarm Assays. Quantitative swarm expansion assays in which mid-log phase cultures were concentrated and used to inoculate swarm agar plates. Swarm expansion was monitored along the same axis every 30 min for 6.5 hrs. Each data point represents the average of three replicates and raw values can be found in Supplementary Table S2. The following strains were used as the inoculum: A) *soe2* (DK3180) and *soe28* (DK6533). B) *soe11* (DK5523), *soe15* (DK5524), *soe26* (DK5525), *soe9* (DK5527), *soe13* (DK5528), and *soe7* (DK5526). C) *soe16* (DK5900), *soe22* (DK5529), *soe23* (DK5531), *soe12* (DK5530), and *soe18* (DK5901). D) *efp nusG* (DK5513). E) *efp flgM* (DK2365), and *efp flgM* P_{IPTG}-*sigD* (DK7073). E) WT (DK1042) and *fliY*^{S164A} (DK6526).
