# Supplementary material for: Suppressor mutations in ribosomal proteins and FliY restore Bacillus subtilis swarming motility in the absence of EF-P
Source: PLoS Genet. 2019 Jun 25;15(6):e1008179. doi: 10.1371/journal.pgen.1008179 (PMC6613710; doi:10.1371/journal.pgen.1008179)
Supplement: S2 Fig — The distribution of homologs of the Soe proteins S10 (dark blue), S3 (black), NusG (red), YeeI (gold), YacO (green), Rae1 (purple), and YdiF (cyan) across the three domains of life. Numbers indicate the following clades (1) Flavobacterium-Cytophaga-Bacteroides group, (2) Chlamydiales, (3) Planctomycetes, (4) Spirochaetes, (5) Actinobacteria, (6) Deinococcus-Thermus group and (7) Cyanobacteria. Bacillus subtilis is highlighted in pink. S7 Table contains the homolog accession numbers for each species. We note that an S3 homolog was not detected in 3 genomes analyzed. Due to the presence of S3 homologs in close relatives of those strains, we predict that this is due to incomplete genome annotation. (PDF) [file pgen.1008179.s004.pdf]

Supplementary Figure 2

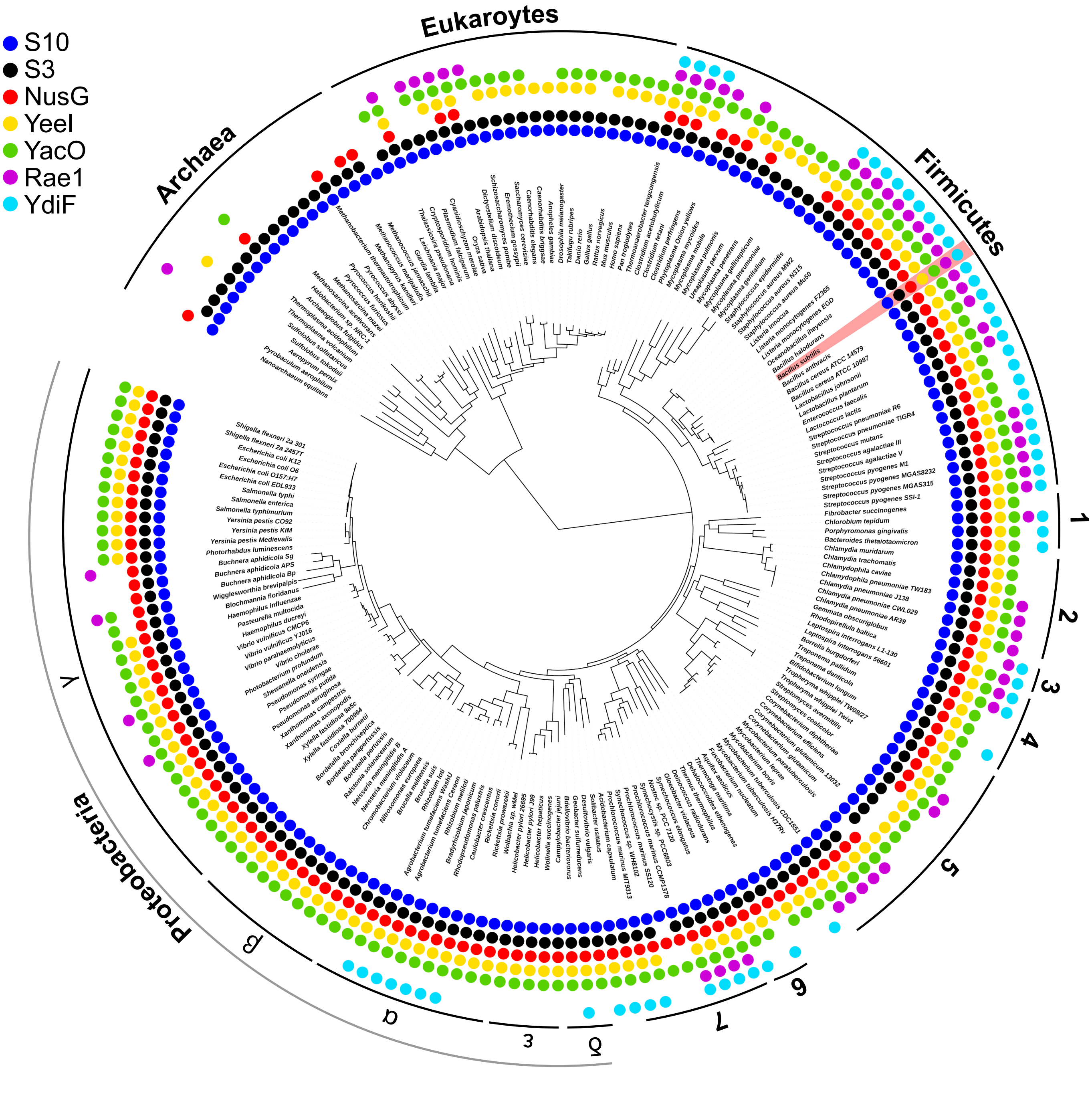

Supplementary Figure 2. Soe proteins are broadly distributed across the tree of life. The distribution of homologs of the Soe proteins S10 (dark blue), S3 (black), NusG (red), YeeI (gold), YacO (green), Rae1 (purple), and YdiF (cyan) across the three domains of life. Numbers indicate the following clades (1) Flavobacterium-Cytophaga-Bacteroides group, (2) Chlamydiales, (3) Planctomycetes, (4) Spirochaetes, (5) Actinobacteria, (6) Deinococcus-Thermus group and (7) Cyanobacteria. Bacillus subtilis is highlighted in pink. Supplementary Table 7 contains the homolog accession numbers for each species. We note that an S3 homolog was not detected in 3 genomes analyzed. Due to the presence of S3 homologs in close relatives of those strains, we predict that this is due to incomplete genome annotation.
