# Supplementary material for: Suppressor mutations in ribosomal proteins and FliY restore Bacillus subtilis swarming motility in the absence of EF-P
Source: PLoS Genet. 2019 Jun 25;15(6):e1008179. doi: 10.1371/journal.pgen.1008179 (PMC6613710; doi:10.1371/journal.pgen.1008179)
Supplement: S3 Fig — β-galactosidase activity reported in Miller Units (MU) of transcriptional fusions of lacZ to PyeeI and PyeeIsoe2. Error bars indicate the standard deviation of 3 biological replicates and the raw values can be found in S2 Table. The following strains were used to generate this panel: PyeeI-lacZ (DK7151), PyeeIsoe2 (DK7152). (PDF) [file pgen.1008179.s005.pdf]

Supplementary Figure 3

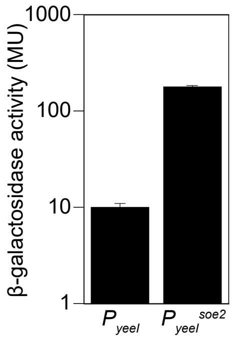

Supplementary Figure 3. *soe2* results in an increase in P_{yeeI} activity. β-galactosidase activity reported in Miller Units (MU) of a transcriptional fusion of *lacZ* to P_{yeeI} and P_{yeeI}^{soe2}. Error bars indicate the standard deviation of 3 biological replicates and raw values can be found in Supplementary Table S2. The following strains were used to generate this panel: P_{yeeI}-*lacZ* (DK7151), P_{yeeI}^{soe2}-*lacZ* (DK7152).
