# Supplementary material for: Suppressor mutations in ribosomal proteins and FliY restore Bacillus subtilis swarming motility in the absence of EF-P
Source: PLoS Genet. 2019 Jun 25;15(6):e1008179. doi: 10.1371/journal.pgen.1008179 (PMC6613710; doi:10.1371/journal.pgen.1008179)
Supplement: S4 Fig — The data used to generate this figure are derived from GEO accession number GSE64488. Panels A, B) Average ribosome profiling pause scores of each codon within the ValS open reading frame. The position of the PPP motif is indicated by a red asterisk on the X-axis. Panels C, D) Weighted sequence logos of amino acid sequences in which the P-site codon had a pause score of 10 or greater in the ribosome profiling datasets from WT or efp. Panels E, F) Average ribosome profiling pause scores of each codon within the FliN open reading frame. The box indicates the location of the VVVV motif. (PDF) [file pgen.1008179.s006.pdf]

Supplementary Figure 4

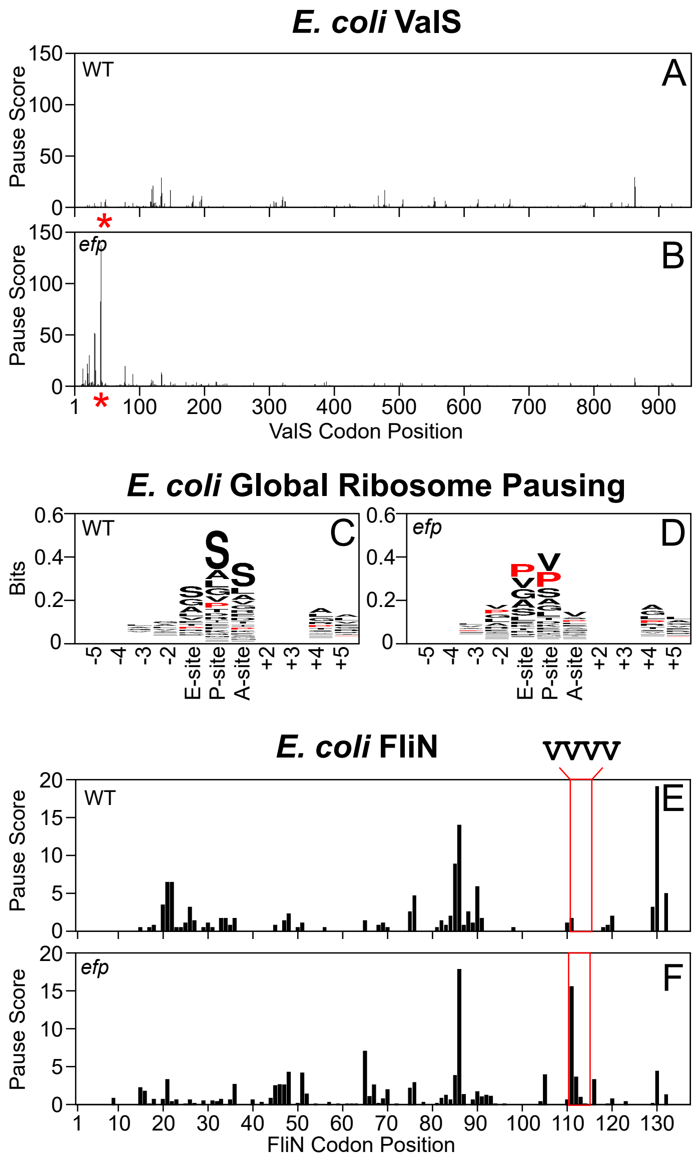

Supplementary Figure 4. *Escherichia coli efp* mutants have increased ribosome pausing in ValS and FliN. The data used to generate this figure are derived from Woolstenhulme et al., 2015. Panels A, B) Average ribosome profiling pause scores of each codon within the ValS open reading frame. The position of the PPP motif is indicated by a red asterisk on the X-axis. Panels C, D) Weighted sequence logos of amino acid sequences in which the P-site codon had a pause score of 10 or greater in the ribosome profiling datasets from WT or *efp*. Panels E, F) Average ribosome profiling pause scores of each codon within the FliN open reading frame. The box indicates the location of the VVVV motif.
