# Supplementary material for: Suppressor mutations in ribosomal proteins and FliY restore Bacillus subtilis swarming motility in the absence of EF-P
Source: PLoS Genet. 2019 Jun 25;15(6):e1008179. doi: 10.1371/journal.pgen.1008179 (PMC6613710; doi:10.1371/journal.pgen.1008179)
Supplement: S5 Fig — Panels A, C) Average ribosome profiling pause scores of each codon within the ValS open reading frame. The position of the PPP motif is indicated by a red asterisk on the X-axis. Panels B, D) Average pause scores for ValS codons 28–58. The box indicates the location of the PPP motif. Error bars indicate standard deviation of 3 biological replicates. The following strains were used to generate this figure: WT (DK1042), and efp (DK2050). (PDF) [file pgen.1008179.s007.pdf]

Supplementary Figure 5

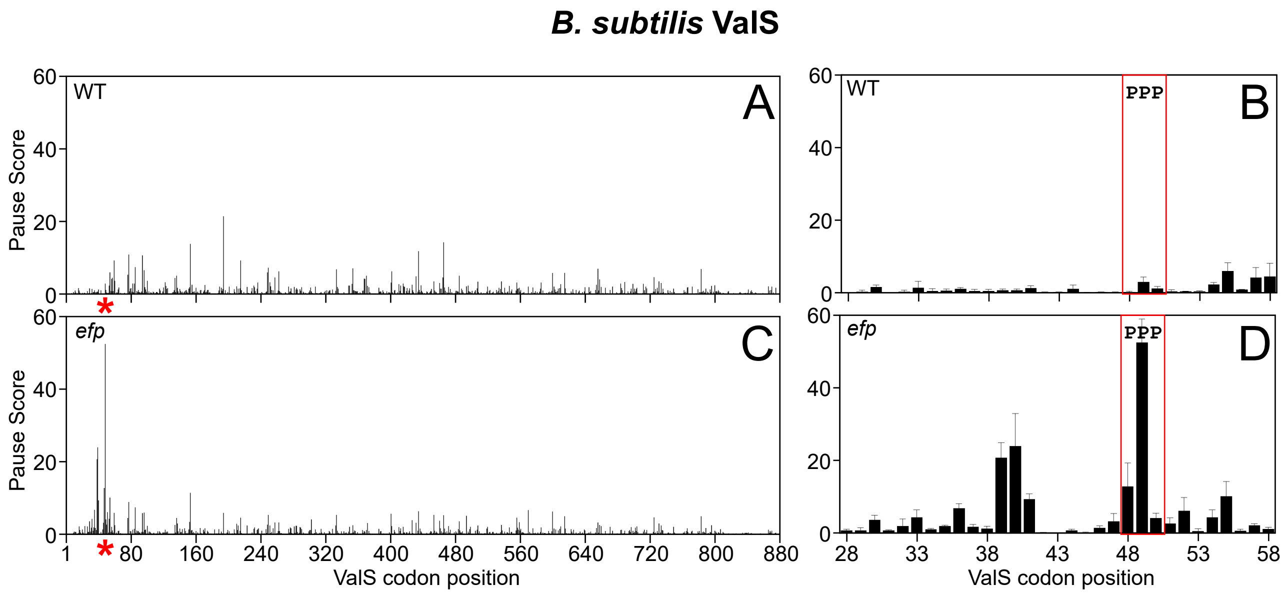

Supplementary Figure 5. EF-P alleviates ribosome pausing in the valine tRNA synthetase, ValS. Panels A, C) Average ribosome profiling pause scores of each codon within the ValS open reading frame. The position of the PPP motif is indicated by a red asterisk on the X-axis. Panels B, D) Average pause scores for ValS codons 28-58. The box indicates the location of the PPP motif. Error bars indicate standard deviation of 3 biological replicates. The following strains were used to generate this figure: WT (DK1042), and *efp* (DK2050).
