# Supplementary material for: Suppressor mutations in ribosomal proteins and FliY restore Bacillus subtilis swarming motility in the absence of EF-P
Source: PLoS Genet. 2019 Jun 25;15(6):e1008179. doi: 10.1371/journal.pgen.1008179 (PMC6613710; doi:10.1371/journal.pgen.1008179)
Supplement: S6 Table — Quantification of the number of occurrences (#) and corresponding percent (%) of each cluster of orthologous groups within genes containing EF-P alleviated ribosome pause sites and all genes encoded on the NCIB 3610 chromosome. (DOCX) [file pgen.1008179.s013.docx]

|  | **EF-P dependent** | | **Total Genome** | |
| --- | --- | --- | --- | --- |
| COG cluster - Description | # | % | # | % |
| C - Energy production and conversion | 7 | 3.9 | 174 | 3.9 |
| D - Cell cycle control, cell division, chromosome partitioning | 3 | 1.7 | 36 | 0.8 |
| E - Amino acid transport and metabolism | 10 | 5.6 | 281 | 6.4 |
| F - Nucleotide transport and metabolism | 2 | 1.7 | 92 | 2.1 |
| G - Carbohydrate transport and metabolism | 12 | 6.7 | 260 | 5.9 |
| H - Coenzyme transport and metabolism | 8 | 4.4 | 110 | 2.5 |
| I - Lipid transport and metabolism | 5 | 2.8 | 87 | 2.0 |
| J - Translation, ribosomal structure and biogenesis | 7 | 3.9 | 163 | 3.7 |
| K - Transcription | 18 | 10 | 290 | 6.6 |
| L - Replication, recombination and repair | 27 | 15.0 | 160 | 3.6 |
| M - Cell wall/membrane/envelope biogenesis | 6 | 3.3 | 214 | 4.8 |
| N - Cell motility | 4 | 2.2 | 40 | 0.9 |
| O - Posttranslational modification, protein turnover, chaperones | 6 | 3.3 | 110 | 2.5 |
| P - Inorganic ion transport and metabolism | 11 | 6.1 | 192 | 4.3 |
| Q - Secondary metabolites biosynthesis, transport and catabolism | 6 | 3.3 | 57 | 1.3 |
| S - Function unknown | 28 | 15.6 | 926 | 20.9 |
| T - Signal transduction mechanisms | 8 | 4.4 | 138 | 3.1 |
| U - Intracellular trafficking, secretion, and vesicular transport | 0 | 0 | 29 | 0.7 |
| V - Defense mechanisms | 1 | 0.6 | 62 | 1.4 |
| Unassigned | 13 | 7.2 | 958 | 21.7 |
