# Supplementary material for: Suppressor mutations in ribosomal proteins and FliY restore Bacillus subtilis swarming motility in the absence of EF-P
Source: PLoS Genet. 2019 Jun 25;15(6):e1008179. doi: 10.1371/journal.pgen.1008179 (PMC6613710; doi:10.1371/journal.pgen.1008179)
Supplement: S9 Table — (DOCX) [file pgen.1008179.s016.docx]

| **Plasmid** | |  | **Genotype** | **Reference** |
| --- | --- | --- | --- | --- |
|  | pKRH9 | | *Ω∆fliMY mls amp* |  |
|  | pKRH20 | | *ΩyeeI^soe2,5,10,19,21,28^ mls amp* |  |
|  | pKRH24 | | *amyE::P_hyspank_-yeeI spec amp* |  |
|  | pKRH25 | | *amyE::P_hyspank_-yeeI^T19K^ spec amp* |  |
|  | pKRH28 | | *ΩyeeI^soe29^ mls amp* |  |
|  | pKRH91 | | *amyE::P_fla/che_-fliY-lacZ spec* |  |
|  | pKRH94 | | *amyE::P_fla/che_-fliY^S164A^-lacZ spec* |  |
|  | pKRH116 | | *amyE::P_hyspank_-nusG spec amp* |  |
|  | pKRH133 | | *ΩyacO^soe11^ mls amp* |  |
|  | pKRH134 | | *ΩyacO^soe15/20^ mls amp* |  |
|  | pKRH135 | | *ΩyacO^soe26^ mls amp* |  |
|  | pKRH136 | | *Ωrae1^soe7^ mls amp* |  |
|  | pKRH137 | | *Ωrae1^soe9^ mls amp* |  |
|  | pKRH138 | | *Ωrae1^soe13^ mls amp* |  |
|  | pKRH139 | | *ΩydiF^soe12^ mls amp* |  |
|  | pKRH140 | | *ΩydiF^soe16^ mls amp* |  |
|  | pKRH141 | | *ΩydiF^soe18^ mls amp* |  |
|  | pKRH142 | | *ΩydiF^soe22^ mls amp* |  |
|  | pKRH143 | | *ΩydiF^soe32^ mls amp* |  |
|  | pKRH144 | | *ΩnusG^N21S^ mls amp* |  |
|  | pKRH145 | | *ΩfliY^S164A^ mls amp* |  |
|  | pKRH165 | | *ΩfliY^S164A^ mls amp* |  |
|  | pKRH180 | | *amyE::P_yeeI_-lacZ cat amp* |  |
|  | pKRH181 | | *amyE::P_yeeI_^soe2^-lacZ cat amp* |  |
|  | pDP288 | | *P_T7_-6-His-SUMO-fliY amp* |  |
|  | pKB43 | | *P_T7_-6-His-SUMO-fliG amp* |  |
|  | pSG6 | | *Ω∆fliY mls amp* | Calvo, 2015 |
|  | pMiniMAD | | *ori^BsTs^ mls amp* | Patrick, 2008 |
|  | pTB146 | | *P_T7_-6-His-SUMO amp* | Bendezύ, 2009 |
|  | pDG268 | | *amyE::lacZ cat amp* | Antoniewski, 1990 |
|  | pDG780 | | *amp kan* | Geurot-Fleury, 1995 |
|  | pDG1515 | | *amp tet* | Geurot-Fleury, 1995 |
|  | pDG1728 | | *amyE::lacZ spec amp* | Guérout-Fleury, 1996 |
|  | pAH54 | | *amp spec* | Geurot-Fleury, 1995 |
