# Supplementary material for: Suppressor mutations in ribosomal proteins and FliY restore Bacillus subtilis swarming motility in the absence of EF-P
Source: PLoS Genet. 2019 Jun 25;15(6):e1008179. doi: 10.1371/journal.pgen.1008179 (PMC6613710; doi:10.1371/journal.pgen.1008179)
Supplement: S10 Table — (DOCX) [file pgen.1008179.s017.docx]

| **Primer** | |  | **Sequence** |
| --- | --- | --- | --- |
|  | 322 | | Ctcctgctagctcagtttttttcaccctcaatatcct |
|  | 869 | | AGGAGgctagcggccgggtgaatcgaaaacagg |
|  | 1295 | | aggaggctcttcaggtatggagaataatagattatctcaag |
|  | 1296 | | ctcctctcgagatctacaattaaaattctatgtgccat |
|  | 1703 | | aggaggtcgacaagaggtgaacaagatggagaataat |
|  | 1704 | | ctcctggatccgtgccataatctatctctctccttg |
|  | 2015 | | aggaggaattcttgctgaccgtgtcggcat |
|  | 3250 | | acgactcactatagggcgaattg |
|  | 3251 | | ctcactaaagggaacaaaagctgg |
|  | 4135 | | gtcgactctagaggatccccTAACCCGTTTGAACGGGCAG |
|  | 4136 | | AATTATTAATGCGCTCTGCCTGGAGAACTTCTCCTGACATTTTC |
|  | 4137 | | GAAAATGTCAGGAGAAGTTCTCCAGGCAGAGCGCATTAATAATT |
|  | 4138 | | gtgaattcgagctcggtacccGCGAAAATCATCTTTACAAAATC |
|  | 4346 | | ATTGATAGAATATGACATATCTCA |
|  | 4347 | | CATGGGCAAAGAATCCTTTAC |
|  | 4349 | | TTGAAGCATGTGCCCGGAG |
|  | 4350 | | TTATGCGAAGTTTGGCCGTG |
|  | 4351 | | GTGAAGTAACGCTTCCGGAT |
|  | 4401 | | gtcgactctagaggatccccGTTCTTGCAGGCCGTGTAC |
|  | 4406 | | AGGAGgaattcTCTTGCTTTACGGAAGACGG |
|  | 4408 | | TCTCCaagcttCATATATATCAATCTAAATACTGTT |
|  | 4409 | | AGGAGgctagccGTGAATAACAGTATTTAGATTGAT |
|  | 4410 | | TCCTCGCATGcGTATGAGAATATCTTATAGATCG |
|  | 4411 | | gtcgactctagaggatccccAAGCGCTCCCGCATTTAGAG |
|  | 4412 | | gtgaattcgagctcggtacccTACCTGCTGAACATCTTCCAA |
|  | 5072 | | CTTCTGCATGCTTAATCTTCTTCTGTGGATAGTT |
|  | 5073 | | GTGAAATTATAGTGAAGAAATGC |
|  | 5074 | | CAATTCGCCCTATAGTGAGTCGTGTTTAAAATAGTATCAGCACCAA |
|  | 5075 | | CCAGCTTTTGTTCCCTTTAGTGAGTGAGAAGCTTAATCAGGAGCT |
|  | 5076 | | CTGGTGGGAGCGCTTCTTA |
|  | 5077 | | tgtgcttgtgcatgacatcat |
|  | 5078 | | caattcgccctatagtgagtcgtccctatgacgtaatcatgttg |
|  | 5079 | | ccagcttttgttccctttagtgaggatgtatgaagtctaccggaaac |
|  | 5080 | | ctcctgaaatgacatccagc |
|  | 5081 | | ggaagaagaggaagcggtg |
|  | 5082 | | caattcgccctatagtgagtcgtcatgttgtacccgtctactaa |
|  | 5083 | | ccagcttttgttccctttagtgagacgtttgaaaagtggaggcg |
|  | 5084 | | acctccacaatttaaaagcgc |
|  | 5085 | | CAGTACGGACAGTCGAAGAT |
|  | 5086 | | TGGCGGCAGCTGACCCG |
|  | 5087 | | CCTCTACCACCAAAAGGGC |
|  | 5088 | | aaagaggaacgacttagtcag |
|  | 5089 | | ttcctgtagcaagagtcacca |
|  | 5090 | | aaatattatacagtattgggcaa |
|  | 5894 | | TCCTCGTCGACAGGAGGTGAAATATCAAtTTTTTT |
|  | 5968 | | tcctttgaattcctacgtctc |
|  | 5969 | | caattcgccctatagtgagtcgtgtacgtgtgaacaacatacca |
|  | 5970 | | ccagcttttgttccctttagtgaggagctggaatttacccaaatc |
|  | 5971 | | caccagcagcttcagcttc |
|  | 5978 | | AGGAGGCTAGCttgaagcgttatggtaattcac |
|  | 5979 | | TCCTCGCATGCggataagtccaagacgtacc |
|  | 6019 | | AGGAGGAATTCttctaagcagcttaacggtct |
|  | 6020 | | TCCTCGGATCCcccgccggcttttcggaa |
|  | 6021 | | AGGAGGGATCCggtcggcgctcatggcat |
|  | 6022 | | gagacgtaggaattcaaagga |
|  | 6023 | | AGGAGGGATCCTATAGCAGAGGTGAAATAATAT |
|  | 6024 | | CTTCTGTCGACAGGAATTCGCGGTGCATATG |
|  | 6025 | | CTTCTGGATCCgattacaatacccatactgcg |
|  | 6446 | | CTTCTGGATCCACTTCCGGCAGAAAGCTCTA |
